# Sex differences in the lipid profiles of visceral adipose tissue with obesity and gonadectomy

**DOI:** 10.1016/j.jlr.2025.100803

**Published:** 2025-04-15

**Authors:** Mita Varghese, Rajendiran Thekkelnaycke, Tanu Soni, Jiayu Zhang, Krishnarao Maddipati, Kanakadurga Singer

**Affiliations:** 1 Department of Pediatrics, Michigan Medicine, University of Michigan, Ann Arbor, MI, USA; 2 Michigan Regional Comprehensive Metabolomics Resource Core, University of Michigan, Ann Arbor, MI, USA; 3 Department of Pathology, Wayne State University, Detroit, MI, USA

**Keywords:** Obesity, metabolism, adipose tissue, fatty acids, phospholipids, prostaglandins, sex hormones, inflammation, lipidomics, sex differences

## Abstract

In obesity, adipose tissue (AT) expansion is accompanied by chronic inflammation. Altered lipid composition in the visceral or gonadal white AT (GWAT) directly drive AT macrophage accumulation and activation to a proinflammatory phenotype. Sex steroid hormones modulate visceral versus subcutaneous lipid accumulation that correlates with metabolic syndrome, especially in men and postmenopausal women who are more prone to abdominal obesity. Prior studies demonstrated sex differences in GWAT lipid species in HFD-fed mice, but the role of sex hormones is still unclear. We hypothesized that sex hormone alterations with gonadectomy (GX) would further impact lipid composition in the obese GWAT. Untargeted lipidomics of obese GWAT identified sex differences in phospholipids, sphingolipids, sterols, fatty acyls, saccharolipids and prenol lipids. Males had significantly more precursor fatty acids (palmitic, oleic, linoleic, and arachidonic acid) than females and GX mice. Targeted lipidomics for fatty acids and oxylipins in the HFD-fed male and female GWAT stromal vascular fraction identified higher omega-6 to omega-3 free fatty acid profile in males and differences in PUFAs-derived prostaglandins, thromboxanes, and leukotrienes. Both obese male and female GWAT stromal vascular fraction showed increased levels of arachidonic acid–derived oxylipins compared to their lean counterparts. Bulk RNA-seq of sorted GWAT AT macrophages highlighted sex and diet differences in PUFA and oxylipin metabolism genes. These findings of sexual dimorphism in both stored lipid species and PUFA-derived mediators with diet and GX emphasize sex differences in lipid metabolism pathways that drive inflammation responses and metabolic disease risk in obesity.

Sex steroid hormones modulate and influence adipose tissue (AT) distribution, fat accumulation, and immune responses in males and females (1, 2). In obesity, central visceral fat accumulation confers a detrimental effect on health (2) as observed in young men, while young and premenopausal women typically have greater peripheral subcutaneous AT accumulation (1, 3, 4). Increased visceral adiposity shifts lipid and glucose metabolism (5, 6) towards an activated immune response characterized by the accumulation and recruitment of AT macrophages (ATM) (7, 8) via production of pro-inflammatory cytokines and chemokines (9, 10). Obesity-induced chronic inflammation promotes AT insulin resistance associated with a broad range of metabolic consequences such as type 2 diabetes, cardiovascular diseases, hypertension, and atherosclerosis that characterize the metabolic syndrome (8, 11, 12). Sex differences in fat accumulation in the AT also lead to sexual dimorphism in immune responses. Our prior work with high fat diet (HFD)-fed obese mouse models demonstrated sex differences in macrophage accumulation and polarization with males having increased circulating monocytes and pro-inflammatory ATMs, contributing to insulin resistance, whereas females are protected from this metabolic inflammation (13, 14, 15). Similarly, postmenopausal women demonstrate gradual increases in visceral adiposity and metabolic complications, eventually reaching or exceeding risk for metabolic disease compared to men (16, 17). This phenomenon suggests an important role for sex hormones in addition to diet in regulating the metabolic fate of nutrients and protecting against metabolic disease pathophysiology.

Previous rodent and human studies with excessive caloric intake suggest that dietary lipids and free fatty acids (FFAs) shape the adipocyte microenvironment and the surrounding immune cell phenotype (9, 18, 19, 20, 21) through mechanisms including ER stress, hypoxia, and cellular senescence (22, 23, 24). Phenotypic switching of macrophages to a proinflammatory state in obesity (25) is affected by surrounding lipid composition and the channeling of exogenous fatty acids into endogenous lipid pools (26, 27, 28), acting as reservoirs for downstream oxylipin production (29, 30). Depending on their acyl chain length, for example, short-, medium-, or long-chain, and saturation status, FAs have wide ranging effects on adipocytes and macrophages. In vitro, uptake of long chain saturated fatty acids (SFAs) such as palmitate in mature adipocytes induced ER stress, increased macrophage chemo-attraction protein-1 (MCP1) and interleukin-6 cytokine levels (31, 32), and also inhibited adipogenesis (33) via detrimental ceramide generation (34) or directly through activation of toll-like receptor 4 pathways (33). In macrophages, palmitate exposure induced mitochondrial dysfunction and apoptosis (35). In contrast, oleic acid, a MUFA, stimulated adipogenesis (36). In adipocytes, dietary PUFAs suppress lipogenesis as they regulate cellular fatty acid levels by inhibiting the liver X receptor-sterol regulatory element-binding protein-1c system (37, 38, 39, 40) as well as act as activators/ligands for the peroxisome proliferator-activated receptors (PPARs) (41, 42). Dietary n-3 PUFAs specifically decrease visceral AT mass and suppress obesity in rodents by targeting transcription factors involved in both adipogenesis and lipid homeostasis (43, 44, 45). The same set of factors can be targeted by n-6 PUFAs but they may exert either an anti- or pro-adipogenic effect depending on experimental settings (46, 47, 48). In other studies, conditioned media from adipocytes exposed to lipopolysaccharide or palmitate promoted a distinct inflammatory response in the bone marrow–derived macrophages (33) showing that adipocyte-derived signals influence the inflammatory status of immune cells. These studies imply a crucial role for dietary fatty acids and their metabolites in the regulation of AT function and inflammation.

Previous studies from our lab with untargeted lipidomics of visceral AT from HFD-fed male and female mice showed that diacylglycerol, ceramides, phospholipids, and certain FA species associated with inflammation were sexually dimorphic with elevated levels in male gonadal WAT (GWAT) than obese female GWAT (15). This suggests a role for sex hormones in sexual dimorphism of lipid mediators in visceral AT in obesity. In the current study, to further investigate the effects of sex hormone manipulations on the lipidome, we applied untargeted lipidomics to visceral AT from HFD-fed castrated and ovariectomized mice. We hypothesized that castration would ameliorate inflammatory lipid mediators, while ovariectomy would elevate those lipid species. Demonstrating the complexity of lipid metabolism and sex, GWAT lipidomics identified shifts in glycerolipids, glycerophospholipids, sphingolipids, sterols, fatty acyls, saccharolipids, and prenol lipids both by sex and hormone status, many of which are implicated in the regulation of inflammation. In addition, males showed elevated levels of key precursor free FAs (palmitic, linoleic, and arachidonic acid) when compared to female and gonadectomized (GX) mice. Targeted lipidomic analysis of GWAT stromal vascular fraction (SVF) PUFA metabolites showed significantly higher ratio of omega 6:omega 3 PUFA substrates in the M HFD SVF fraction demonstrating sex differences in PUFA metabolism. Comparisons of a dietary challenge on oxylipin derivatives with targeted lipidomics in male and female GWAT SVF with and without HFD feeding demonstrated a sexually dimorphic pattern in the mediators that incite an inflammatory response. Obese male GWAT SVF showed a larger proportion of significantly upregulated oxylipins than lean male and obese female. The proportion of arachidonic acid (AA)-derived metabolites that were significantly different were also higher in obese male GWAT SVF than obese female oxylipins that preferred linoleic acid (LA) as their precursor when compared to lean female GWAT SVF. Altogether, our data suggest that both hormone status and diet influence sex-specific alterations in GWAT lipid storage and metabolism that may drive sexually dimorphic ATM polarization accompanied by sex differences in oxylipin production.

## Materials and Methods

### Animals

C57Bl/6J (000664) gonadectomized (GX) and Sham surgery animals were purchased from Jackson Laboratories at 4 weeks of age. Mice were gonadectomized at 3–4 weeks of age. All mice were fed ad libitum either a normal diet (ND, control) consisting of standard chow, 13.5% fat (5LOD; LabDiet), or HFD consisting of 60% of calories from fat (Research Diets; D12492), starting at 6 weeks of age for 16 weeks of duration. Animals were housed in a specific pathogen-free facility with a 12-h light/12-h dark cycle and given free access to food and water. Animal protocols were in compliance with the Institute of Laboratory Animal Research Guide for the Care and Use of Laboratory Animals and approved by the University Committee on Use and Care of Animals at the University of Michigan (animal welfare assurance number: A3114-01).

### Untargeted lipidomics—liquid chromatography with tandem mass spectrometry

For untargeted lipidomic studies, GWAT from six mice in each group—male sham HFD, female sham HFD, male castrated/gonadectomized (M GX) HFD, and female ovariectomized/gonadectomized (F GX) HFD were submitted to the Metabolomics Core at University of Michigan.

#### Reagents and internal standards

High performance liquid chromatography (HPLC)-grade acetonitrile and dichloromethane were purchased from Sigma-Aldrich, isopropanol [Optima; liquid chromatography with mass spectrometry (LC-MS) grade] was purchased from Thermo Fisher Scientific, and methanol (LC-MS grade) was from J.T. Baker. Water was obtained from a high-purity water dispenser (EMD Millipore). The following MS–grade lipid standards were obtained from Sigma-Aldrich: 1-heptadecanoyl-2-hydroxy-sn-glycero-3- phosphocholine (17:0/0:0), 1,2-diheptadecanoyl-sn-glycero-3-phosphocholine (17:0/17:0), 1,2-diheptadecanoyl-sn-glycero-3-phosphoethanolamine (17:0/17:0), 1,2-diheptadecanoylsn-glycero-3-phospho-L-serine (sodium salt) (17:0/17:0), N-heptadecanoyl-D-erythro-sphingosylphosphorylcholine 17:0 (d18:1/17:0), cholest-5-en-3b-yl heptadecanoate 17:0 cholesteryl ester, 1-palmitoyl-2-oleoyl-sn-glycerol 16:0-18:1, 1-heptadecanoyl-rac-glycerol 17:0, 1,2,3-triheptadecanoyl-glycerol triheptadecanoate 17:0, N-heptadecanoyl-D-erythro-sphingosine C17 (d18:1/17:0), 1,2-diheptadecanoyl-sn-glycero-3-phosphate (sodium salt) 17:0, 1,2-diheptadecanoyl-sn-glycero-3-phospho- (10-rac-glycerol) (sodium salt) 17:0, 1-heptadecanoyl-2- (5Z,8Z,11Z,14Z eicosatetraenoyl)-sn-glycero-3-phospho-(10-myoinositol) (ammonium salt) 17:0-20:4, 1,3-Dinonadecanoyl-rac-glycerol, 1,3-Dinvvonadecanoylglycerol,-(19:0/0:0/19:0), and glyceryl tri (palmitated31) d31. More information about internal standards is provided in supplemental Table S1.

#### Sample preparation for untargeted lipidomics

GWAT was excised at the end of 16 weeks of HFD. The tissues were accurately weighed and then homogenized. Lipids were extracted using a modified Bligh-Dyer method (49) using a 2:2:2 ratio volume of methanol/water/dichloromethane at room temperature after spiking internal standards (described in supplemental Table S1) The organic layer was collected and completely dried under nitrogen. Before MS analysis, the dried lipid extract was reconstituted in 100 μl of buffer B (10:85:5 acetonitrile/isopropyl alcohol/water) containing 10 mM ammonium acetate and subjected to LC-MS.

#### Internal standards and quality controls

Quality control samples were prepared by pooling equal volumes of each sample and injected at the beginning and the end of each analysis and after every 10 sample injections to provide a measurement of the system’s stability and performance as well as reproducibility of the sample preparation method (50). Two kinds of controls were used to monitor the sample preparation and MS. To monitor instrument performance, 10 μl of a dried matrix-free mixture of the internal standards reconstituted in 100 μl of buffer B (85% isopropyl alcohol/10% acetonitrile/5% water in 10 mM NH4OAc) was analyzed. As additional controls to monitor the profiling process, an equimolar mixture of 13 authentic internal standards (15) and a characterized pool of human plasma and test pool (a small aliquot from the all-AT samples used in this study; extracted in tandem with tissue samples) were analyzed along with AT samples. Each of these controls was included several times into the randomization scheme such that sample preparation and analytical variability could be monitored constantly.

#### Data-dependent LC-MS/MS for the measurements of lipids

Chromatographic separation was performed on a Shimadzu CTO-20A Nexera X2 UHPLC system equipped with a degasser, binary pump, thermostat autosampler, and column oven [all components manufactured by Shimadzu]. The column heater temperature was maintained at 55°C and an injection volume of 5 ml was used for all analyses. For lipid separation, the lipid extract was injected onto a 1.8-mm particle diameter, 50-mm 3 2.1-mm inner diameter Waters Acquity HSS T3 column (Waters). Elution was performed using acetonitrile/water (40:60, v/v) with 10 mM ammonium acetate as solvent A and acetonitrile/water/isopropanol alcohol (10:5:85, v/v/v) with 10 mM ammonium acetate as solvent B. For chromatographic elution, we used a linear gradient during a 20-min total run time, with a 60% solvent A and 40% solvent B gradient in the first 10 min. Then the gradient was ramped in a linear fashion to 100% solvent B, which was maintained for 7 min. Thereafter, the system was switched back to 60% solvent B and 40% solvent A for 3 min. The flow rate used for these experiments was 0.4 ml/min and the injection volume was 5 ml. The column was equilibrated for 3 min before the next injection and run at a flow rate of 0.400 ml/min for a total run time of 20 min. MS data acquisition for each sample was performed in both positive and negative ionization modes using a TripleTOF 5600 equipped with a DuoSpray ion source (AB Sciex). Column effluent was directed to the electrospray ionization source and voltage was set to 5500 V for positive ionization and 4500 V for the negative ionization mode. The declustering potential was 60 V and the source temperature was 450 °C for both modes. The curtain gas flow, nebulizer, and heater gas were set to 30, 40, and 45, respectively (arbitrary units). The instrument was set to perform one time of flight MS survey scan (150 ms) and 15 MS/MS scans with a total duty cycle time of 2.4 s. The mass range of both modes was 50–1,200 m/z. Acquisition of MS/MS spectra was controlled by the data-dependent acquisition function of the Analyst TF software (AB Sciex) with application of the following parameters: dynamic background subtraction, charge monitoring to exclude multiply charged ions and isotopes, and dynamic exclusion of former target ions for 9 s. Collision energy spread of 20 V was set whereby the software calculated the collision energy value to be applied as a function of m/z. A DuoSpray source coupled with automated calibration system (AB Sciex) was used to maintain mass accuracy during data acquisition. Calibrations were performed at the initiation of each new batch or polarity change.

All lipids were identified using MS/MS fragmentation (precursor m/z range 200–1,200) following separation by RP-UPLC-ESI-QTOF-MS/MS. For initial assignment of lipid identities, we used the “LipidBlast” spectral library coupled with NIST spectral search tools, which match compounds by observed mass and MS/MS fragmentation pattern. To facilitate accurate lipid identification, a stringent mass error tolerance of 0.001 m/z for positive mode and 0.005 m/z for negative mode were used. These values were determined based on the observed mass accuracy of internal standards and were selected to reduce the rate of false positive identifications. Annotations were validated using authentic standard compounds for all major lipid classes. MS/MS data for annotation and authentic standards from major lipid classes was used to distinguish accurate annotations from incorrect assignments of identities of adducts. Chromatographic retention time was also used as a means of validating lipid identifications. Lipids of a particular class can be expected to follow a regular order of elution, wherein chromatographic retention time should increase with increasing alkyl chain length and decrease with increasing degree of desaturation (number of double bonds); all lipid identifications were checked to ensure lipids within a class adhere to this pattern.

#### Data processing

The raw data were converted to mgf data format using Proteo Wizard software (51). The NIST MS PepSearch program was used to search the converted files against LipidBlast (52, 53) libraries in batch mode. We optimized the search parameters using the NIST11 library and LipidBlast libraries and comparing them against our lipid standards. The m/z width was determined by the mass accuracy of internal standards and was set at 0.001 for the positive mode and 0.005 for the negative mode. The minimum match factor used in the PepSearch Program was set to 200. The MS/MS identification results from all of the files were combined using an in-house software tool to create a library for quantification. All raw data files were searched against this library of identified lipids with mass and retention time using MultiQuant 1.1.0.26 (AB Sciex) (54, 55). Quantification was done using MS1 data. The quality control samples were also used to remove technical outliers and lipid species that were detected below the lipid class–based lower limit of quantification. Quality control samples evenly distributed along analytical runs of the study were analyzed. The average coefficient of variation of all the lipids detected in the study samples was 25%.

Raw data from untargeted lipidomics are deposited at the Metabolomics Workbench (study_id: ST003785, Project DOI: 10.21228/M89Z6M).

### Targeted lipidomics for oxylipins—High performance liquid chromatography

#### Adipose tissue SVF isolation

AT fractionation was performed as described previously (14). Briefly, whole GWAT pads were minced and digested with type II collagenase (Sigma 1 mg/ml in RPMI media) for 15–30 min at 37 °C on a rocker. Filtrated samples were spun at 500g for 10 min and RBC lysis was conducted (biosciences 00-4333-57). After wash, the pelleted SVF cells were counted for normalization and were saved in PBS at −20°C prior to oxylipin processing.

#### Sample preparation for targeted lipidomics

For targeted lipidomic studies, GWAT SVF from six mice in each group—male normal diet (M ND), male HFD (M HFD), female normal diet (F ND), and female HFD (F HFD) were submitted to the Lipidomics Core at Wayne State University. Samples (adjusted to a maximum volume of 1–2 ml) were spiked with a mixture of internal standards consisting of 15(S)-HETE-d8, Leukotriene B4-d4, Resolvin D2-d5, 14(15)-EpETrE-d11, and prostaglandin E1-d4 (5 ng each) for recovery and quantitation and mixed thoroughly. The samples were then extracted for PUFA metabolites using C18 extraction columns as described earlier (56, 57, 58, 59). Briefly, the internal standard spiked samples were applied to conditioned C18 cartridges, washed with water followed by hexane and dried under vacuum. The cartridges were eluted with 0.5 ml methanol. The eluate was dried under a gentle stream of nitrogen. The residue was redissolved in 50 μl methanol-25 mM aqueous ammonium acetate (1:1) and subjected to LC-MS analysis.

#### HPLC and data processing

HPLC was performed on LC40D XR system (Sciex) using Luna C18 (3μ, 2.1 × 150 mm) column. The mobile phase consisted of a gradient between A: methanol-water-acetonitrile (10:85:5 v/v) and B: methanol-water-acetonitrile (90:5:5 v/v), both containing 0.1% ammonium acetate. The gradient program with respect to the composition of B was as follows: 0–1 min, 50%; 1–8 min, 50%–80%; 8–15 min, 80%–95%; and 15–17 min, 95%. The flow rate was 0.2 ml/min. The HPLC eluate was directly introduced to OptiFlowPro ESI source of QTRAP7500 mass analyzer (SCIEX) in the negative ion mode with following conditions: curtain gas and GS1 at 40 psi and GS2: 70 psi, temperature: 600 °C, ion spray voltage: −2500 V, collision gas: low, declustering potential: −60 V, and entrance potential: −7 V. The eluate was monitored by multiple reaction monitoring (MRM) method to detect unique molecular ion–daughter ion combinations for each of the 125 transitions (to monitor a total of 156 lipid mediators). The MRM was scheduled to monitor each transition for 120 s around the established retention time for each lipid mediator. Optimized collisional energies (18–35 eV) and collision cell exit potentials (7–10 V) were used for each MRM transition. Mass spectra for each detected lipid mediator were recorded using the enhanced product ion feature to verify the identity of the detected peak in addition to MRM transition and retention time match with the standard. The data was collected using SciexOS 3.3 software, and the MRM transition chromatograms are quantitated by SciexOS Analytics software (both from SCIEX). The internal standard signals in each chromatogram were used for normalization for recovery as well as relative quantitation of each analyte. All the oxylipins were stable under the analytical methods used (21).

### Adipose tissue SVF isolation and flow cytometry

AT fractionation and flow cytometry analysis were performed as described previously (13). Briefly, whole AT was minced and digested with type II collagenase (Sigma; 1 mg/ml in RPMI media) for 15–30 min at 37 °C on a rocker. Samples were filtered and spun at 500g for 7 min, and red blood cell lysis was conducted (Biosciences; 00-4333-57). SVF cells were stained with anti-mouse CD45 eFluor450 (30-F11 monoclonal; Invitrogen), CD64 PE (X54-5/7.1 monoclonal; BD Pharmingen), and CD11c eFluor 780 (N418 monoclonal; Invitrogen). Gating was performed for macrophage populations by CD45 gates to determine ATMs (14). Lipid-Tox Deep Red (Invitrogen) was added to detect lipid uptake in the ATMs.

### Quantitative real-time PCR

RNA was extracted from AT SVF using the RNeasy Kit for cells (Qiagen). Complementary DNA was generated using a High-Capacity cDNA Reverse Transcription Kit (Applied Biosystems). SYBR Green PCR Master Mix and the StepOnePlus System (Applied Biosystems) were used for real-time quantitative PCR. *Arbp* expression was used as an internal control for data normalization. Samples were assayed in duplicate, and relative expression was determined using the 2−ΔΔCT method. All primers used are listed in the supplemental Table S2.

### RNA-sequencing

In this previously published dataset, the RNA-seq experiment consisted of four groups with four replicates per group; groups were defined by gender (M/F) and diet (ND/HFD) (60). Following cell sorting, ATM cells were pelleted, and RNA was prepared using a Qiagen RNA kit. As standard practice in the sequencing core, quality control was performed for concentration and RNA integrity number. After quality control, library preparation and sequencing was also performed at the University of Michigan Advanced Genomics Core. Libraries were prepared with the Takara/Clonetech SMARTer standard kit, after ribodepletion with RiboGene, resulting in 125 base fragments. Sequencing was performed on the Illumina Hi-Seq 4000 platform, with 50 single-end cycles. Fastq read files were uploaded for analysis. Data analysis was performed by the University of Michigan Bioinformatics Core. FastQC (version v0.11.3) was used for quality control before and after alignment, and all samples passed. The Tuxedo Suite was used for alignment, differential expression analysis, and post-analysis diagnostics (61, 62, 63). TopHat (version 2.0.13) and Bowtie2 (version 2.2.1) were used for alignment based on the UCSC mm10 reference. Cuttlinks/Cutt Diff (version 2.1.1) was used for expression quantitation, normalization, and differential expression analysis. To identify a given gene as differentially expressed, the cutoffs were as follows: absolute fold change of at least 1.5, and false discovery rate (FDR) corrected *P*-value less than or equal to 0.05. Data are publicly available on NCBI GEO accession GSE181841.

### Statistical analysis

The sample peak intensities were normalized through internal standards. For untargeted lipidomics data, PCA was performed by combining the positive and negative mode datasets and a sample correlation matrix was generated to detect and omit sample outliers (supplemental Fig. S1A, B). Thereafter, further analysis was performed with six samples in M Sham HFD and five samples each in the other groups including F Sham HFD, M GX HFD, and F GX HFD GWAT. Further data processing for both untargeted and targeted lipidomics was performed in MetaboAnalyst 6.0. The quality of preprocessed data was improved with the robust interquantile range method to filter 25% of relative standard deviation in variables. Data normalization was performed through pareto scaling and log transformation (base 10). For two-group comparisons in MetaboAnalyst, univariate analysis with the Student’s *t* test and variable fold change analysis with an FDR of 0.05 was conducted. Differences with a *P* ≤ 0.05 were considered significant. Volcano plots were created to visualize significant lipids satisfying both FDR (<0.05) and fold change (>2) for both untargeted and targeted lipidomic data. Bar graphs were analyzed by 2-way ANOVA interaction accounting for main effects of sex and gonadectomy (GX) or sex and diet followed by posthoc analysis for multiple comparisons by Fisher’s LSD test in GraphPad Prism 10.2.

## Results

Recent studies from our lab showed that ovariectomized mice had increased weight gain, adiposity, increased ATMs, and bone marrow myeloid colonies compared with Sham-gonadectomized females (64). In addition, castrated males exposed to HFD had improved glucose tolerance, insulin sensitivity, and adiposity with fewer Ly6c^hi^ monocytes and bone marrow myeloid colonies although AT macrophages remained elevated (64). Sex differences in inflammatory responses suggest alterations in lipid storage and metabolism dependent on sex hormone manipulations. To further investigate the sex differences in lipidome changes in the adipose that can affect ATM responses, we performed untargeted lipidomics with GWAT from castrated and ovariectomized (gonadectomized/GX) mice on HFD.

### Sex and sex hormone differences in total lipids in GWAT

With untargeted lipidomics, a total of 666 lipids were identified in the GWAT samples analyzed from M Sham HFD, F Sham HFD, M GX HFD, and F GX HFD groups (see Statistical analysis of lipidomic data in Materials and Methods). Table 1 depicts the major lipid class and subclasses detected in the GWAT of all the groups. In addition, ether-linked lipids (TG-O, PE-O, PC-O, LPE-O) were also detected, although it was not possible to distinguish plasmanyl from plasmenyl lipid species.

**Table 1:** Lipid classes and subclasses detected by untargeted lipidomics in the GWAT of M Sham HFD, M GX HFD, F Sham HFD, and F GX HFD groups

Lipid Class	Subclass	Abbreviation
Glycerolipid		GL
	Triglyceride	TG
	Diglyceride	DG
Glycerophospholipid		GPL
	Bis-mono-oleoylglycerophosphate	BMP
	Phosphatidylethanolamine	PE
	Phosphatidic acid	PA
	Phosphatidylcholine	PC
	Phosphatidylinositol	PI
	Phosphatidylserine	PS
	Phosphatidyl-methanol	PMeOH
	Phosphatidyl-ethanol	PMetOH
Lysophospholipid	Lyso-Phosphatidylcholine	LPC
	Lyso-Phosphatidylethanolamine	LPE
	N-acyl-Lysophosphatidylethanolamine	LNAPE
Ether-linked/Plasmanyl	Triglyceride-O	TG-O
	Phosphoethanolamine-O	PE-O
	Phosphatidylcholine-O	PC-O
	Lyso-phosphatidylethanolamine-O	LPE-O
Sphingolipid		SP
	Ceramide	Cer
	Dihexosyl-Cer	Hex2-Cer
	PE-Cer	PE-Cer
	Sphingomyelin	SM
Sterol		ST
	Cholesterol	
	Cholesterol ester	CE
Saccharolipid		
	Monogalactosyldiacylglycerol	MGDG
	Diacylglyceryl glucuronide	ADGGA
Fatty acyls		
	Fatty acids	FA
	Acylcarnitine	CAR
	Fatty acyl ester of hydroxy FA	FAHFA

N = M Sham HFD (6), F Sham HFD (5), M GX HFD (5), and F GX HFD (5). F, female; GWAT, gonadal white adipose tissue; GX, gonadectomy; HFD, high-fat diet; M, male.

**Fig. 1:**
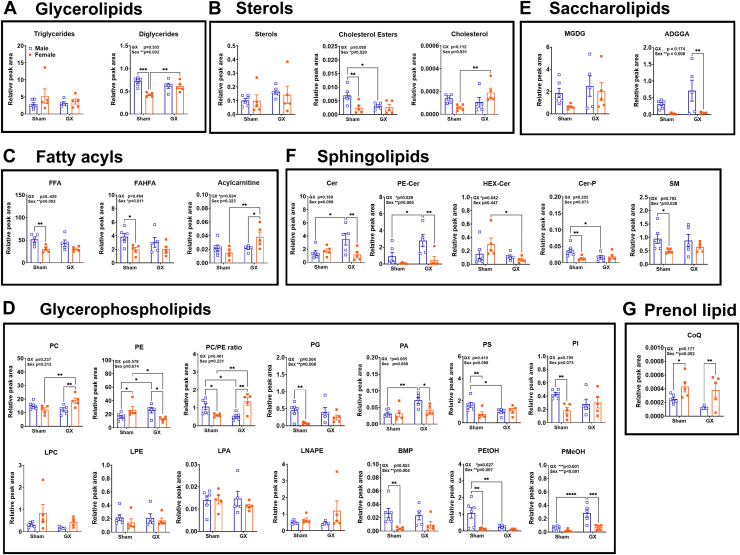
Lipid compositions from untargeted lipidomics in the GWAT of male (M) and female (F) mice in response to HFD and GX. Plots depicting relative abundance between M SHAM HFD, F SHAM HFD, M GX HFD, and F GX HFD GWAT as peak area of (A) glycerolipids, (B) sterols, (C) fatty acyls, (D) glycerophospholipids, (E) saccharolipids, (F) sphingolipids, (G) prenol lipid. Data analysis was performed by 2-way ANOVA accounting for sex and GX followed by *post hoc* analysis for multiple comparisons with Fisher’s LSD test. Data shown as average ± SEM. ∗*P* < 0.05, ∗∗*P* < 0.01, ∗∗∗*P* < 0.001, and ∗∗∗∗*P* < 0.0001. N = M Sham HFD (6), F Sham HFD (5), M GX HFD (5), and F GX HFD (5). GWAT, gonadal white adipose tissue; HFD, high-fat diet; GX, gonadectomy.

**Fig. 2:**
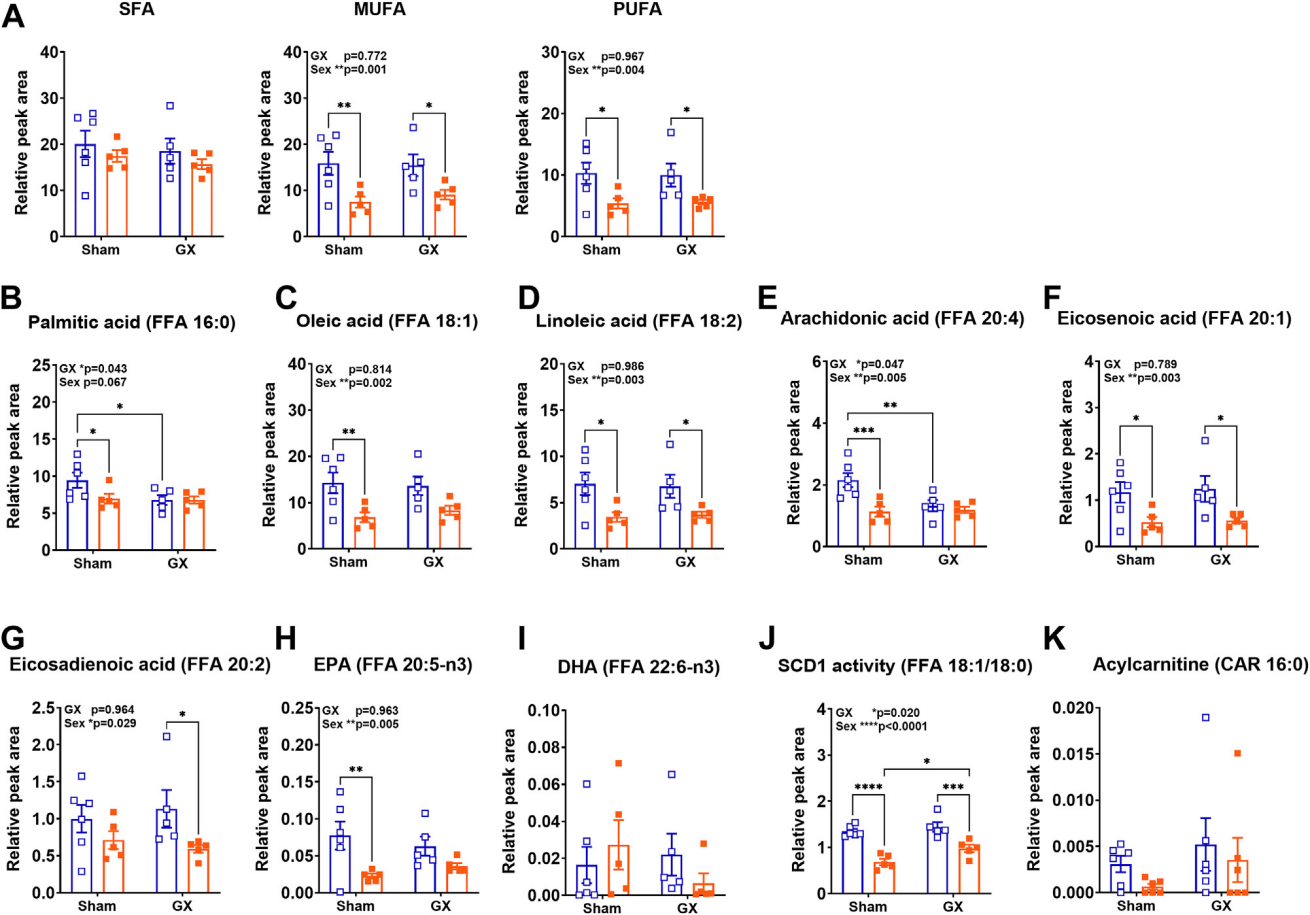
FA compositions in the GWAT of male (M) and female (F) mice in response to HFD and GX from untargeted lipidomics. Plots depicting relative abundance as peak area of (A) SFA (*left*), MUFA (*middle*), and PUFA (*right*), (B) palmitic acid, (C) oleic acid, (D) linoleic acid, (E) arachidonic acid, (F) eicosenoic acid, (G) eicosadienoic acid, (H) EPA, (I) DHA, (J) SCD1 activity, (K) acylcarnitine. Data analysis was performed by 2-way ANOVA accounting for sex and GX followed by *post hoc* analysis for multiple comparisons with Fisher’s LSD test. Data shown as average ± SEM. ∗*P* < 0.05, ∗∗*P* < 0.01, ∗∗∗*P* < 0.001, and ∗∗∗∗*P* < 0.0001. N = M Sham HFD (6), F Sham HFD (5), M GX HFD (5), and F GX HFD (5). GWAT, gonadal white adipose tissue; SFA, saturated fatty acid; MUFA, mono-unsaturated fatty acid; PUFA, poly-unsaturated fatty acid; HFD, high-fat diet; GX, gonadectomy; EPA, eicosapentaenoic acid; DHA, docosahexaenoic acid; SCD1, stearoyl-CoA desaturase 1.

Relative quantification of different lipid subclasses in obese GWAT demonstrated significant changes in several lipid classes by sex and GX status (Fig. 1). Diglycerides (DGs) were increased significantly in M Sham HFD and F GX HFD GWAT compared to F Sham HFD (Fig. 1A), while triglycerides were unchanged. Sterol content did not alter significantly among the groups, but cholesterol ester (CE) content was specifically decreased in F Sham HFD GWAT and in M GX GWAT compared to M Sham HFD GWAT (Fig. 1B). Total cholesterol levels were significantly higher in F GX HFD GWAT than F Sham (Fig. 1B). DGs and CE relative levels were significantly different for main effect of sex (*P* < 0.05 and *P* < 0.01). Among the fatty acyls, FFA and FAHFA content was higher in the M Sham HFD GWAT than F Sham HFD and did not show any changes with GX (Fig. 1C). FFA and FAHFA were changed significantly by sex (*P* < 0.05 and *P* < 0.01). Acylcarnitine levels, however, increased with F GX HFD compared to F Sham HFD GWAT and M GX HFD GWAT but showed no differences between M and F Sham HFD GWAT (Fig. 2C). Thus, acylcarnitine content was significantly different by GX (*P* < 0.05), suggesting insufficient β-oxidation upon GX compared to Sham GWAT, especially in females.

Lipidomic analysis revealed characteristic differences in cellular phospholipids. Within glycerophospholipids, PC content was higher with F GX HFD compared to M GX HFD and F Sham HFD GWAT (Fig. 1D). Phosphatidylethanolamine (PE) content remained higher with M GX HFD and in F Sham GWAT than M Sham, while showing lower levels in F GX HFD GWAT than F Sham, but M GX HFD PE content was higher than F GX HFD group (Fig. 1D). In the obese GWAT, PC/PE ratio was higher in females than males, but castration decreased the ratio while ovariectomy increased it (Fig. 1D, top panel), suggesting a lipidome remodeling of PC and PE species after GX. Prostaglandin (PG) levels were significantly lower in F Sham GWAT than males, while GX showed no changes (Fig. 1D). M GX HFD GWAT had increased phosphatidic acid (PA) levels than M Sham and F GX HFD (Fig. 1D). Phosphatidylserine (PS) and phosphatidylinositol levels remained higher in M Sham and did not increase in F Sham or with GX in either sex (Fig. 1D). No significant changes were observed in lyso-phosphatidylcholine (LPC), lyso-phosphatidylethanolamine (LPE), lyso-phosphatidic acid, or N-acetyl LPE (LNAPE) (Fig. 1D, bottom panel). Bis-mono-oleoylglycerophosphate (BMP) content was higher in M Sham than other groups (Fig. 1D). PEtOH levels were significantly higher in M Sham than M GX HFD and F Sham GWAT (Fig. 1D). Male PMeOH content increased with GX and was significantly higher than F GX HFD GWAT (Fig. 1D). PG and BMP were different by sex (*P* < 0.05 and *P* < 0.01), while PA content was significant by GX (*P* < 0.05). PMeOH and PEtOH content were significantly different by both sex (*P* < 0.01 and *P* < 0.001) and GX (*P* < 0.01 and *P* < 0.001) implying a tighter control by sex hormones and sex on its regulation (*P* < 0.001).

For saccharolipids, monogalactocyldiacylglycerol was not significantly different among groups, but diacylglyceryl glucoronide content was lower in F GX HFD GWAT than M GX HFD GWAT (Fig. 1E). Diacylglyceryl glucoronide was significant for main effect of sex (*P* < 0.01) only. Among the sphingolipids, Cer and PE-Cer content increased with M GX and remained higher than F GX HFD GWAT (Fig. 1F). Hex-Cer levels decreased with F GX, while Cer-P were decreased with M GX (Fig. 1F). Cer-P levels were also significantly lower in the F Sham GWAT than M Sham (Fig. 1F). PE-Cer was significant by both sex and FX (*P* < 0.05 and *P* < 0.01), while HEX-Cer was significant by GX (*P* < 0.05) and sphingomyelin (SM) by sex (*P* < 0.05). Among prenol lipids, CoQ levels were higher in F GX than M GX GWAT (Fig. 1G). Also, CoQ was significantly different by sex (*P* < 0.01).

### Sex differences in saturated and unsaturated FA and FA species in GWAT

Next, we investigated within the free FA content, if there are differences in the percentage of SFA and unsaturated FA (UFA) including MUFA and PUFA among the different groups. Figure 2A shows that the percentage of SFA remained similar among the groups with no significant changes. MUFA and PUFA remained similar between M Sham and M GX HFD groups but were reduced in F Sham and F GX groups (Fig. 2A). These results suggest sex differences in the unsaturation of FA in the obese GWAT with males showing more UFA than females, while gonadectomy failed to reverse this dimorphism.

FAs such as palmitic acid (SFA), oleic (MUFA), and linoleic acid (PUFA) were comparatively higher in M Sham than F Sham (Fig. 2B–D). Palmitic acid was significant by GX (*P* < 0.05), while oleic and linoleic acid were significant by sex (*P* < 0.01). Notably, palmitic acid and AA decreased significantly with castration in males (Fig. 2B, E). Proinflammatory PUFA such as AA was also higher in M Sham than F Sham and was significant by sex and GX (*P* < 0.05 and *P* < 0.01) (Fig. 2E). Other PUFAs such as eicosenoic and EPA were also significantly higher in M Sham than F Sham (Fig. 2F, H), while eicosenoic acid and eicosadienoic acid were significantly higher in M GX HFD GWAT than F GX GWAT (Fig. 2F, G). Eicosenoic, eicosadienoic, and EPA were significant by sex (*P* < 0.05 and *P* < 0.01). DHA did not show any considerable differences among the groups (Fig. 2I).

Stearoyl-CoA desaturase 1 (SCD1) is a key enzyme that desaturates the SFAs derived from de novo lipogenesis or diet to generate MUFAs (65). In addition to lower unsaturated fatty acid levels in female, the desaturase activity index of SCD1 calculated by the ratio of stearic acid/oleic acid (18:1/18:0) depicted increased SCD1 activity in the M Sham GWAT compared with F Sham GWAT (Fig. 2J). However, upon ovariectomy, the F GX GWAT responded with increased SCD1 activity compared to F Sham (Fig. 2J). This may suggest a general lower absorption of FFAs in females thereby generating fewer toxic lipid intermediates. Overall, M GX GWAT showed increased SCD1 activity than F GX GWAT (Fig. 2J). SCD1 activity was significant for main effects of sex and GX (*P* < 0.05 and *P* < 0.01). Long-chain acylcarnitine such as CAR 16:0 is implicated in inflammation due to incomplete β-oxidation (66). Since total acylcarnitine showed main effect of GX (Fig. 1C), we investigated CAR 16:0 that trended to be higher in M Sham GWAT than F Sham GWAT and also with GX in M GX GWAT compared to M Sham GWAT, but this was not statistically significant (Fig. 2K).

### Changes in sphingolipid species by sex and with gonadectomy

Sphingolipids such as SM and Cer can affect adipose metabolism (67). SM accumulation has been found to potentially reduce the mitochondrial thermogenic capacity and disrupt proton leakage across the mitochondrial membrane in rodent subcutaneous white AT (67). In humans, circulating levels of SM species with distinct saturated acyl chains (C18:0, C20:0, C22:0, and C24:0) were found to be closely correlated with the parameters of obesity, insulin resistance, liver function, and lipid metabolism in young obese adults (68). Activation of Toll-like receptor 4 by SFA, as well as by independently acting pro-inflammatory cytokines, can lead to upregulation of the Cer biosynthesis pathway. Another pathway to produce Cer is through hydrolysis of SM by acid or neutral sphingomyelinase (69). Cer can decrease insulin sensitivity and glucose transport via activation of PKB (also known as Akt) dephosphorylation by protein phosphatase 2a (67, 70). In human subjects, Cer species (C14:0, C16:0, C16:1, C18:0, C18:1, and C22:1) were found to be elevated in obese AT and positively correlated with insulin resistance (70, 71, 72).

**Fig. 3:**
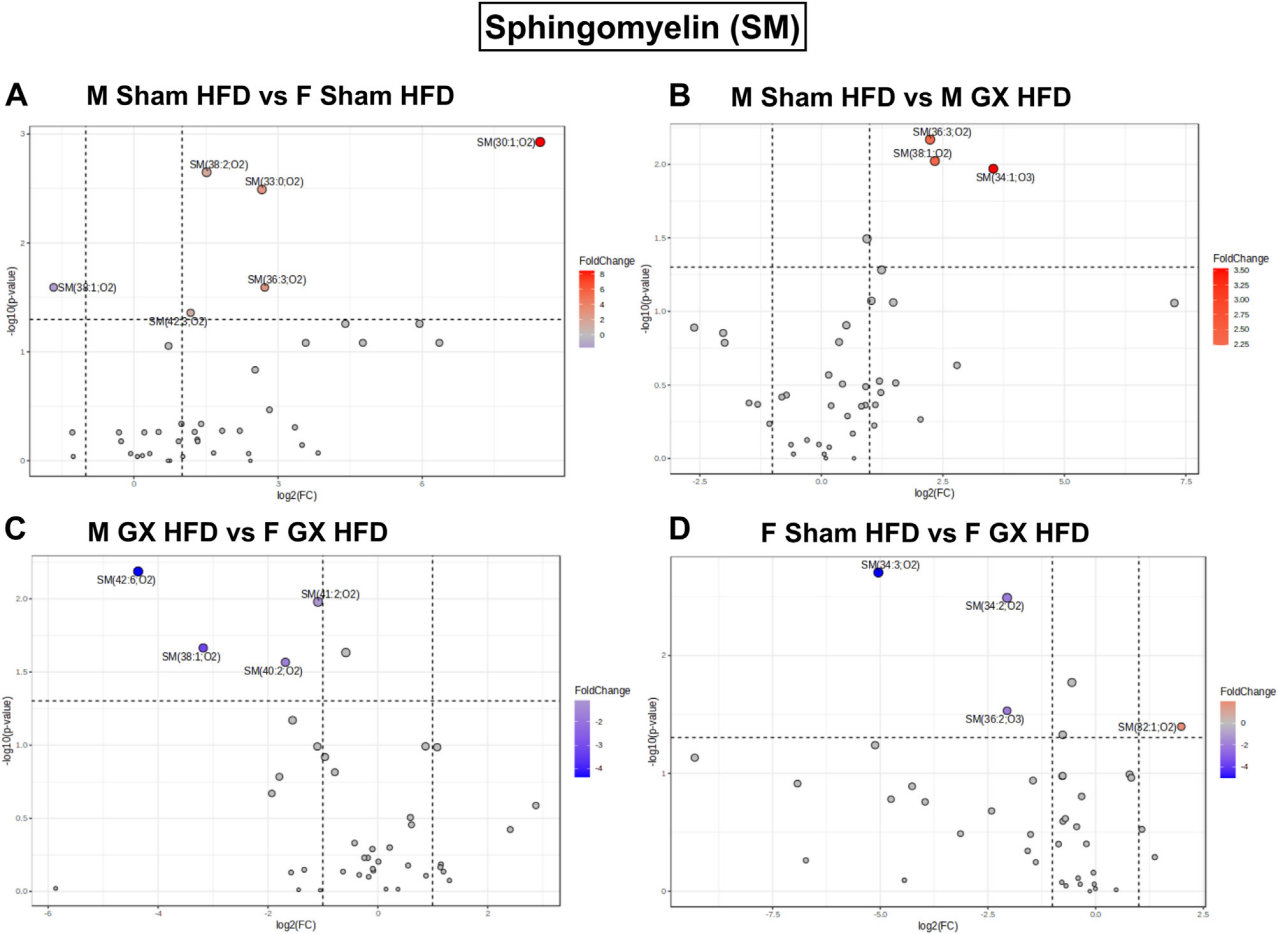
SM species significantly upregulated or downregulated with HFD and GX from untargeted lipidomics. Volcano plots depicting log2 fold changes in SM lipid species and the corresponding significance values displayed as -log10 (*P* value) in (A) M Sham HFD versus F Sham HFD, (B) M Sham HFD versus M GX HFD, (C) M GX HFD versus F GX HFD, and (D) F Sham HFD versus F GX HFD. Each dot represents a lipid species, and the dot size and color intensity indicate significance. *Red* dots represent significantly upregulated and *blue* dots represent significantly downregulated lipid species. Only lipids with *P* < 0.05 and fold change >2 are displayed. N = M Sham HFD (6), F Sham HFD (5), M GX HFD (5), and F GX HFD (5). SM, sphingomyelin; HFD, high-fat diet; GX, gonadectomy.

SM and Cer being bioactive lipid mediators capable of modifying AT metabolism, we sought to further understand the changes in different species of Cer and SM with gonadectomy. We generated volcano plots of significantly regulated species by sex and GX status (Fig. 3). Figure 3A shows significant upregulation of SM(30:1;O2), SM(33:0;O2), SM(36:3;O2), SM(38:2;O2), and SM(42:3;O2) species in M Sham HFD GWAT when compared to F Sham HFD GWAT with the downregulation of SM(38:1;02) (Fig. 3A). In M GX HFD GWAT, SM(36:3;O2), SM(38:1;O2), and SM(34:1;O3) were downregulated when compared to M Sham HFD (Fig. 3B). When compared to F GX HFD GWAT, M GX HFD GWAT showed downregulation of SM(38:1;O2), SM(40:2;O2), SM(41:2;O2), and SM(42:6;O2) (Fig. 3C). In the F Sham HFD GWAT, comparatively, SM(32:1;O2) was significantly upregulated while SM(34:3;O2), SM(34:2;O2), and SM(36:2;O3) were downregulated (Fig. 3D).

SM (36:3;O2) showed a recurrence in Fig. 3A, B, suggesting its significance in the M Sham lipidome compared to F Sham and M GX GWAT SM species (main effect of sex, *P* < 0.05; supplemental Fig. S2A). The commonality of SM (38:1;O2) between Figure 3A–C suggests an effect of the female sex and sex hormones on this SM species, given its downregulation with ovariectomy (F GX) (main effect of sex and GX, *P* < 0.05 and *P* < 0.01; supplemental Fig. S2B).

**Fig. 4:**
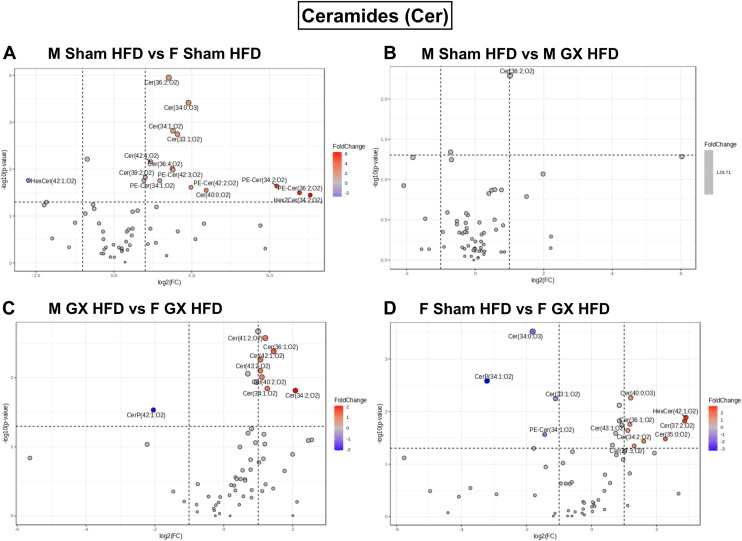
Cer species significantly upregulated or downregulated with HFD and GX from untargeted lipidomics. Volcano plots depicting log2 fold changes in Cer lipid species and the corresponding significance values displayed as -log10 (*P* value) in (A) M Sham HFD versus F Sham HFD, (B) M Sham HFD versus M GX HFD, (C) M GX HFD versus F GX HFD, and (D) F Sham HFD versus F GX HFD. Each dot represents a lipid species, and the dot size and color intensity indicate significance. Only lipids with *P* < 0.05 and fold change >2 are displayed. N = M Sham HFD (6), F Sham HFD (5), M GX HFD (5), and F GX HFD (5). Cer, ceramide; HFD, high-fat diet; GX, gonadectomy.

Among the ceramides (Cer), we identified the biggest upregulation of specific Cer species in M Sham HFD (Fig. 4A–C). Volcano plots showed the upregulation of 14 species in male HFD compared to female HFD fed mice comprising of Cer(36:2;O2), Cer(34:0;O3), Cer(34:1;O2), Cer(33:1;O2), Cer(42:4;O2), Cer(36:4;O2), PE-Cer(42:3;O2), Cer(39:2;O2), PE-Cer(34:1;O2), PE-Cer(34:2;O2), PE-Cer(42:2;O2), Cer(40:0;O2), PE-Cer(36:2;O2), and Hex2Cer(34:2;O2). On the other hand, only one species, HexCer(42:1;O2) was found to be significantly downregulated in M Sham GWAT compared to F Sham GWAT (Fig. 4A). In M GX HFD GWAT, only Cer(36:2;O2) was significantly downregulated while being upregulated in M Sham GWAT (Fig. 4B). Interestingly, when compared to F GX HFD GWAT, M GX HFD GWAT still showed significant upregulation of seven species, Cer(41:2;O2), Cer(36:1;O2), Cer(42:1;O2), Cer(43:2;O2), Cer(40:2;O2), Cer(34:1;O2), and Cer(34:2;O2) and downregulation of CerP(42:1;O2) species (Fig. 4C). F Sham HFD GWAT showed significant upregulation of Cer(40:0;O3), HexCer(42:1;O2), Cer(37:2;O2), Cer(36:1;O2), Cer(43:1;O2), Cer(35:0;O2), Cer(34:2;O2), and Cer(37:3;O2), while Cer(34:0;O3), CerP(34:1;O2), Cer(33:1;O2), and PE-Cer(34:1;O2) were downregulated when compared to F GX HFD GWAT (Fig. 4D) demonstrating the influence of sex and gonad status on ceramides.

Interestingly, the most upregulated number of Cer species were observed with M Sham GWAT when compared to F Sham GWAT (Fig. 4A) and with F GX when compared to F Sham HFD (Fig. 4D) and M GX HFD (Fig. 4C). From the volcano plots, Cer (33:1;O2), Cer (34:1;O2), and Cer (36:2;O2) remained higher in M HFD GWAT compared to F HFD (main effects of sex, *P* < 0.01 and *P* < 0.0001; supplemental Fig. S2C, D, G). Castration did not bring out any significant decline among the Cer species except Cer (36:2;O2) (Fig. 4B and supplemental Fig. S2G, main effect of sex and GX, *P* < 0.05 and *P* < 0.001), suggesting minimum effects on Cer remodeling, but F GX showed significant decreases in Cer (34:2;O2) and Cer (36:1;O2) (main effect of sex, *P* < 0.001; supplemental Fig. S2E, F).

### Global lipidomic analysis shows sex differences in phospholipid species in AT lipid remodeling with gonadectomy

**Fig. 5:**
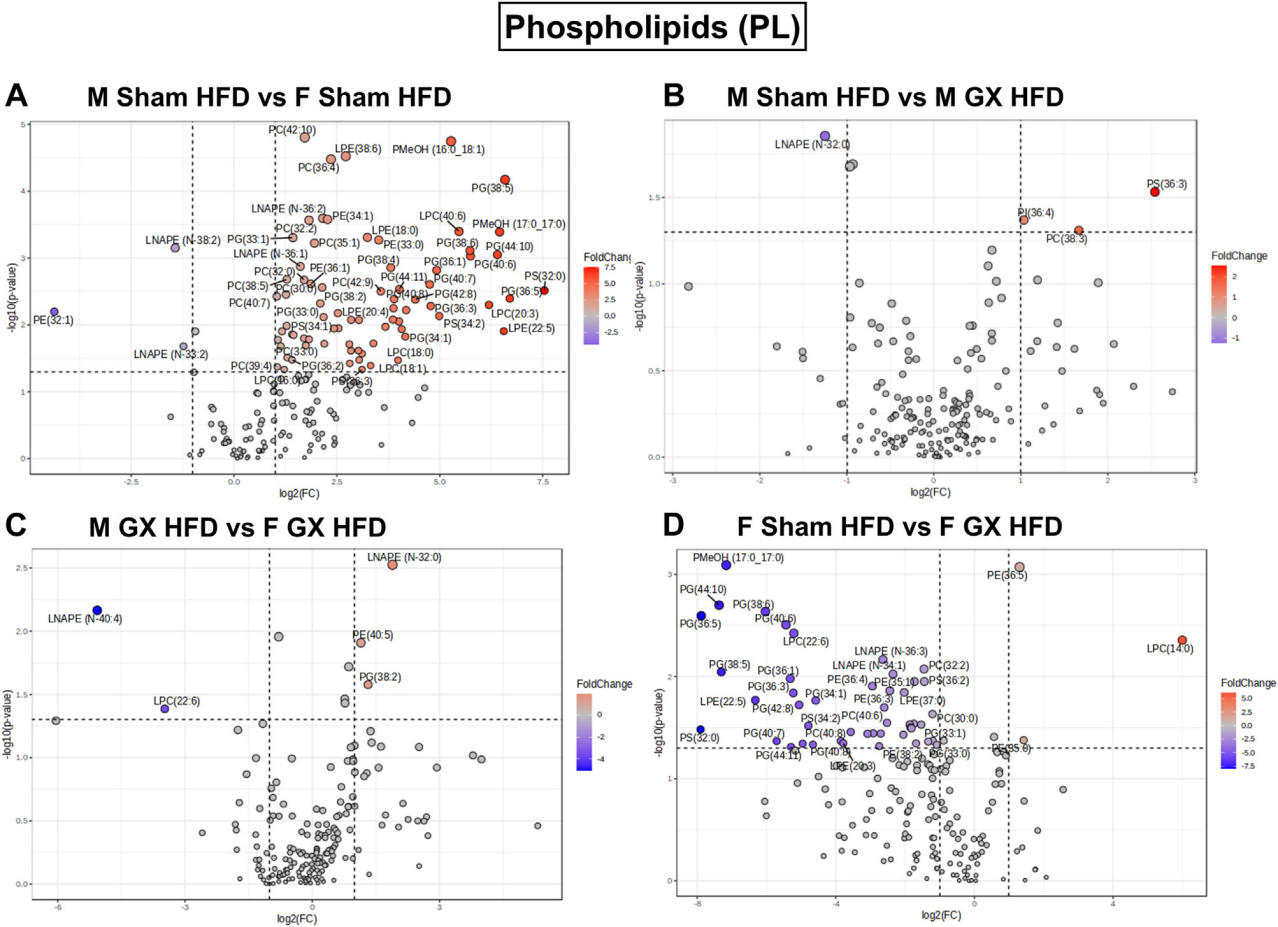
PL species significantly upregulated or downregulated with HFD and GX from untargeted lipidomics. Volcano plots depicting log2 fold changes in PL lipid species and the corresponding significance values displayed as -log10 (*P* value) in (A) M Sham HFD versus F Sham HFD, (B) M Sham HFD versus M GX HFD, (C) M GX HFD versus F GX HFD, and (D) F Sham HFD versus F GX HFD. Each dot represents a lipid species, and the dot size and color intensity indicate significance. Only lipids with *P* < 0.05 and fold change >2 are displayed. N = M Sham HFD (6), F Sham HFD (5), M GX HFD (5), and F GX HFD (5). PL, phospholipid; HFD, high-fat diet; GX, gonadectomy.

We observed an effect of both sex and GX on total glycerophospholipids in the AT (Fig. 1D). To further understand the effect of sex hormone changes on different phospholipid (PL) species, we generated volcano plots for group comparisons that satisfy both FDR (<0.05) and fold change (>2) between M Sham HFD versus F Sham HFD, M Sham HFD versus M GX HFD, F Sham HFD versus F GX HFD, and M GX HFD versus F GX HFD (Fig. 5A–D). Figure 5A shows 75 significantly upregulated PL species (in red) in M Sham HFD GWAT compared to F Sham HFD, while only three species, PE (32:1), LNAPE (N-33:2), and LNAPE (N-38:2) were downregulated in M Sham HFD GWAT compared to female. Compared to M GX HFD GWAT, M Sham HFD GWAT showed significant changes in phosphatidylinositol (36:4), PC (36:3), and PC (38:3) that were upregulated, while LNAPE (N-32:0) was downregulated (Fig. 5B). Between M GX and F GX HFD GWAT, M GX GWAT showed significantly upregulated species of PE (40:5) and PG (38:2) and LNAPE (N-32:0), while LPC (22:6) and LNAPE (N-40:4) were downregulated (Fig. 5C). Interestingly, F Sham GWAT when compared to F GX GWAT showed PE (35:0), PE (36:5), and LPC (14:0) to be upregulated, while 46 PL species were significantly downregulated (Fig. 5D).

Overall, sex differences were observed with significant changes in many PL class profiles between M and F Sham GWAT. Also, F Sham GWAT showed interesting PL differences when compared to F GX GWAT, but not as many significant changes were observed between M Sham GWAT and M GX GWAT. These differences suggest adipose PL remodeling being more prominent with ovariectomy rather than castration with sex playing a larger role in PL species.

### Sex differences in ether-linked PL or plasmalogen profile in GWAT with gonadectomy

Ether lipids are a unique class of peroxisome-derived glycero- and glycerophospho-lipid. They carry an ether or vinyl ether linked fatty alcohol at the sn-1 position, and an ester linked fatty acid either at the sn-2 position (ether phospholipids), or at both the sn-2 and sn-3 positions (ether glycerolipids) (73). Among various lipid species, ether linked PL (plasmalogen, PL-O) also known as plasmalogens are a major subcomponent of adipocyte membrane PL that are involved in the generation of lipid mediators (74, 75).

**Fig. 6:**
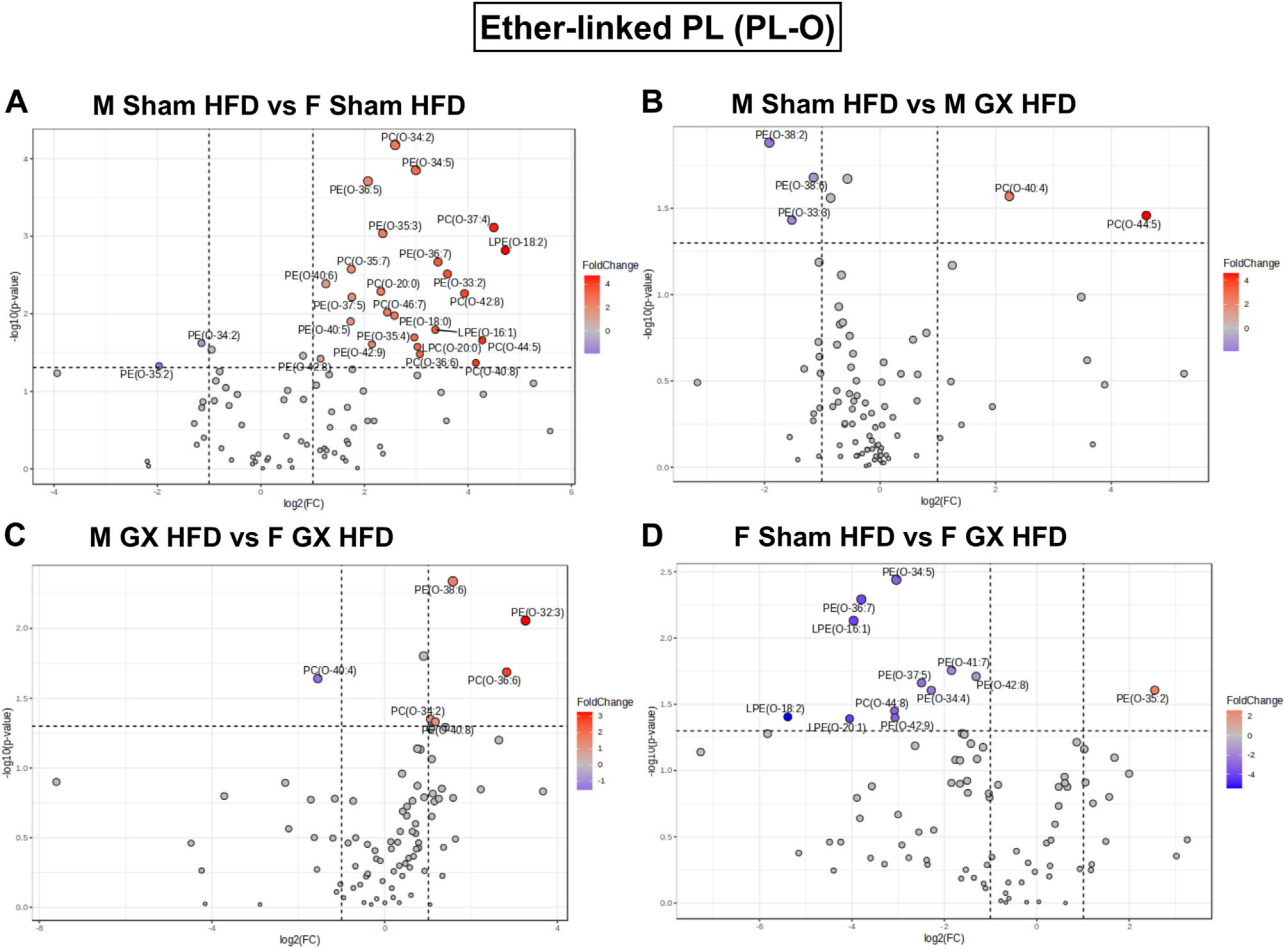
PL-O species significantly upregulated or downregulated with HFD and GX from untargeted lipidomics. Volcano plots depicting log2 fold changes in SM lipid species and the corresponding significance values displayed as -log10 (*P* value) in (A) M Sham HFD versus F Sham HFD, (B) M Sham HFD versus M GX HFD, (C) M GX HFD versus F GX HFD, and (D) F Sham HFD versus F GX HFD. Each dot represents a lipid species, and the dot size and color intensity indicate significance. Only lipids with *P* < 0.05 and fold change >2 are displayed. N = M Sham HFD (6), F Sham HFD (5), M GX HFD (5), and F GX HFD (5). PL-O, Ether-phospholipid; HFD, high-fat diet; GX, gonadectomy.

To further understand the effect of sex hormone changes on ether-linked PL species, we generated volcano plots for group comparisons between M Sham HFD versus F Sham HFD, M Sham HFD versus M GX HFD, F Sham HFD versus F GX HFD, and M GX HFD versus F GX HFD (Fig. 6A–D). The biggest changes were observed between M HFD versus F HFD GWAT showing upregulation of 24 PL-O species including PC-O, PE-O, LPC-O, and LPE-O, while PE-O(34:2) and PE-O(35:2) were downregulated (Fig. 6A). In the M GX HFD GWAT, PE-O(33:3), PE-O(38:2), and PE-O(38:6) were upregulated, while PC-O(40:4) and PC-O(44:5) were downregulated when compared to M HFD GWAT (Fig. 6B). M GX HFD GWAT showed significantly upregulated species of PC-O(34:2), PC-O(36:6), PE-O(32:3), PE-O(38:6), and PE-O(40:8), while PC-O(40:4) was downregulated (Fig. 6C). F Sham HFD GWAT showed significantly higher PE-O(35:2) species when compared to F GX HFD GWAT, while 11 species were downregulated (Fig. 6D). Interestingly, sex differences between M Sham HFD and F Sham HFD GWAT were observed as LPC-O and LPE-O species were upregulated only in the M HFD group (Fig. 6A).

Between the plots, Fig. 6A, D, PE-O(34:5) was higher in M Sham HFD GWAT and increased with ovariectomy than F Sham HFD GWAT (Diet and sex interaction, *P* < 0.05; supplemental Fig. S2H). Notably, a decrease was observed in species PE-O(35:2) with ovariectomy compared to F Sham HFD GWAT (Diet and sex interaction, *P* < 0.05; supplemental Fig. S2I).

### AT macrophage lipid uptake correlates with sex differences in inflammation

**Fig. 7:**
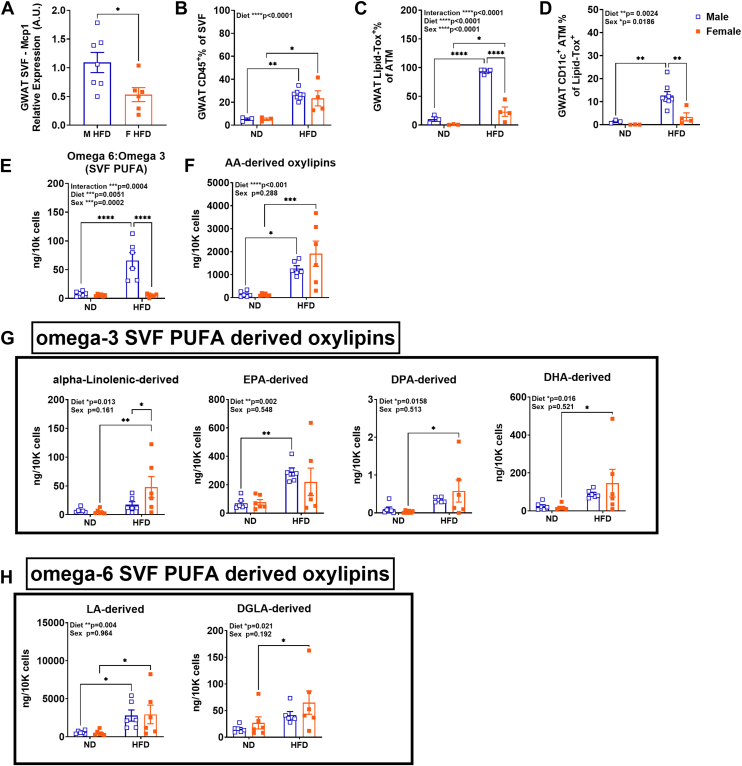
Gene expression, flow cytometry assessment of ATMs, and omega-3 and omega-6 derived oxylipins (targeted lipidomics) in lean and obese GWAT SVF. (A) Mcp1 gene expression in M and F HFD SVF. Quantitation of (B) GWAT CD45+% of SVF, (C) GWAT Lipid-Tox^+^% of ATM, (D) GWAT CD11c^+^ ATM% of Lipid-Tox^+^. N = 3/ND group, N = 4–8/HFD group; data were analyzed by 2-way ANOVA followed by Tukey’s multiple comparisons test. Data shown as average ± SEM. ∗*P* < 0.05, ∗∗*P* < 0.01, ∗∗∗*P* < 0.005, ∗∗∗∗*P* < 0.0001. Plots depicting relative abundance of oxylipins as ng/10k cells of the (E) ratio of omega 6: omega 3 PUFAs (F), arachidonic acid (AA)-derived (G) Omega-3: alpha-linolenic-derived, EPA-derived, DPA-derived, DHA-derived (H) omega-6: LA-derived, DGLA-derived. Data analysis was performed by 2-way ANOVA accounting for sex and diet followed by post hoc analysis for multiple comparisons with Fishers LSD test. Data shown as average ± SEM. ∗*P* < 0.05, ∗∗*P* < 0.01, ∗∗∗*P* < 0.001, and ∗∗∗∗*P* < 0.0001. N = M ND (6), M HFD (6), F ND (6), and F HFD (6). GWAT, gonadal white adipose tissue; SVF, stromal vascular fraction; HFD, high-fat diet; AA, arachidonic acid; EPA, eicosapentaenoic acid; DPA, docosapentaenoic acid; DHA, docosahexaenoic acid, LA, linoleic acid; DGLA, dihomo-gamma-linoleic acid.

In the earlier observations, it was evident that HFD played a major role in bringing out sex differences in the stored fatty acids, especially UFA (MUFA and PUFA) that increased in obese GWAT of males than females, while GX did not reverse the effects significantly (Fig. 2A). Previous studies have also demonstrated sex differences in inflammatory responses with increased inflammation observed in obese male AT than female AT with HFD feeding (13, 14, 15). HFD diet feeding and lipid storage in the AT could have a notable impact on ATM inflammatory response as they constitute majority of the immune cells in the adipose SVF fraction. To investigate this, we performed gene expression of inflammatory gene, *Mcp1*, which was observed to be significantly higher in M HFD SVF than F HFD SVF (Fig. 7A). To investigate whether the increased inflammatory tone in M HFD GWAT corresponds to sex differences in significant lipid uptake by ATMs, we performed flow cytometry analysis of Lipid-TOX in isolated SVF cells from ND and HFD-fed male and female GWAT. Percentage of CD45^+^ leukocytes was higher in both male and female GWAT SVF with HFD feeding (Fig. 7B). We observed a significantly greater percentage of ATMs (CD64^+^CD11c^+^) in male SVF as previously described (14, 64). Lipid-Tox^+^ (Fig. 7C) staining confirmed fewer lipid-Tox^+^ ATMs and pro-inflammatory CD11c^+^ ATMs in obese females than obese males (Fig. 7D). These results suggest a role for HFD in driving inflammation especially in male GWAT ATMs.

### Sex differences in PUFAs give rise to sex differences in PUFA-derived oxylipins and eicosanoids

To investigate sex differences in the PUFA lipid content in GWAT SVF, we performed targeted free fatty acid evaluations in GWAT SVF. Given changes that would occur from dietary challenge based on prior work, we isolated GWAT SVF from both male and female mice on ND and HFD. A significantly higher ratio of omega 6: omega 3 PUFA substrates were observed in the M HFD SVF fraction compared to M ND and F HFD groups (Fig. 7E), demonstrating sex differences in PUFA metabolism. To further understand the effect of PUFA content on macrophage inflammatory response, we further probed the SVF fraction of GWAT from male and female ND and HFD for oxylipin mediators using targeted lipidomics. The three classes of PUFA-derived oxylipins, PGs, leukotrienes (LT), and thromboxanes (TX) as well as resolvins, (Rv), protectins (PD), and maresins (MaR) were evaluated. AA-derived oxylipins were differential by diet (*P* < 0.0001) (Fig. 7F). Total content of all omega-3 SVF PUFA-derived oxylipins (Fig. 7G) and omega-6 SVF PUFA-derived oxylipins (Fig. 7H) were also differential by diet. Notably, omega-3–derived metabolites were increased in F HFD GWAT SVF such as alpha-linolenic and DHA-derived (Fig. 7G), while males appeared to only have an increase in EPA-derived oxylipins (Fig. 7G). F HFD GWAT SVF also showed increase in omega-6–derived metabolites such as LA-derived and dihomo-gamma-LA (DGLA)-derived, while males showed only increase in LA-derived metabolites (Fig. 7H).

**Fig. 8:**
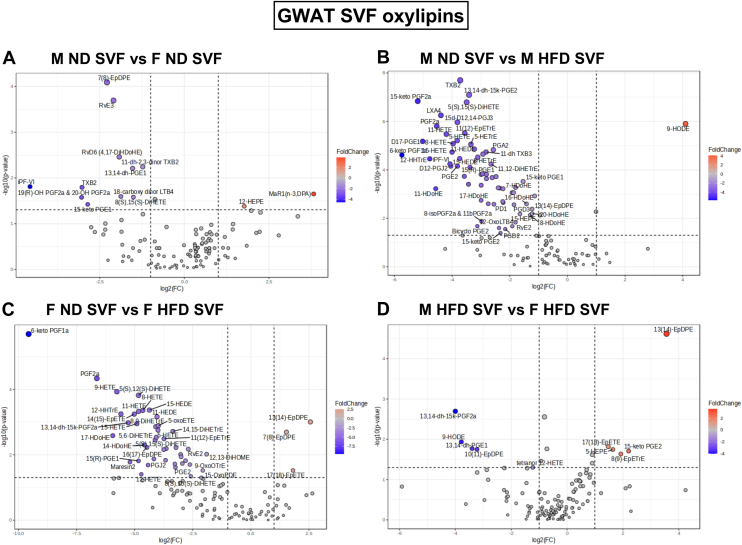
Sex differences in oxylipin lipid alterations in GWAT SVF with ND and HFD from targeted lipidomics. Volcano plots depicting log2 fold changes in oxylipin lipid species and the corresponding significance values displayed as -log10 (*P* value) in (A) M ND versus F ND, (B) M ND versus M HFD, (C) F ND versus F HFD, and (D) M HFD versus F HFD. Each dot represents a lipid species, and the dot size and color intensity indicate significance. Only lipids with *P* < 0.05 and fold change >2 are displayed. N = 6/group. GWAT, gonadal white adipose tissue; SVF, stromal vascular fraction; M, male; F, female; ND, normal diet; HFD, high-fat diet.

We next examined the individual oxylipins to investigate any sex differences in the generation of these metabolites. Cyclooxygenase (COX) or the prostaglandin synthase genes produce PGs that are substrates for a series of downstream enzymes that generate specific PGs, that is, PGE2, PGI2, PGD2, PGF2, and TXA2 (76). Lipoxygenase produce hydroperoxy-eicosatetraenoic acid and hydroxyeicosatetraenoic acids (HETEs) that are converted to LTs, lipoxins (LXs), and hepoxilins including resolvins (Rvs), protectins (PDs), and maresins (MaRs) derived from various omega-3 PUFAs. Cytochromes P450 produce epoxyeicosatrienoic acids, HETEs, epoxyeicosatetraenoic acids (EpETEs), hydroxyeicosapentaenoic acids (HEPEs), epoxydocosapentaenoic acids, and hydroxydocosahexaenoic acids (76). Among the eicosanoid classes, we generated volcano plots to identify significantly upregulated oxylipins between the GWAT SVF groups from M ND, F ND, M HFD, and F HFD (Fig. 8). Between M and F ND SVF, M ND showed significantly higher levels of EPA-derived 12-HEPE (hydroxy-EPA) and docosapentaenoic acid (DPA)-derived MaR1(n-3, DPA). On the other hand, F ND showed higher levels of 11 eicosanoids including AA-derived iPF-VI, 19(R)-OH PGF2a & 20-OH PGF2a, TXB2, 18-carboxy dinor LTB4, 8(S),15(S)-DiHETE, 11-dh-2,3-dinor TXB2; DGLA-derived 15-keto PGE1 and 13,14-dh-PGE1, DHA-derived 7(8)-EpDPE and RvD6; and EPA-derived RvE3 (Fig. 8A). Between M ND and M HFD SVF, M ND SVF showed higher levels of 9-hydroxyoctadecadienoic acid (9-HODE), while M HFD showed higher levels of total 63 eicosanoid species; 35 species of which were derived from AA followed by 13 derivatives of DHA and few of EPA, DGLA, and eicosadienoic acid derivatives (Fig. 8B). Comparison of F ND and F HFD SVF showed that F ND had significant higher levels of epoxy-DHA derivatives, 7(8)-EpDPE, 13(14)-EpDPE, and 17(18)-EpETE derived from EPA while F HFD SVF showed significant higher levels of 40 eicosanoid species of which 21 species were AA-derived while among the rest, six species were DHA-derived, three species were alpha-Linolenic–derived, while two species were LA-derived and the others were EPA, DGLA, and eicosadienoic acid derivatives (Fig. 8C). Between M HFD SVF and F HFD SVF, M HFD showed significantly higher levels species of AA-derived 17(18)-EpETE, 8(9)-EpETrE, and 15-keto PGE2; DHA-derived 13(14)-EpDPE; and EPA-derived 5-HEPE, while F HFD SVF showed higher levels of AA-derived 13,14-dh-15k-PGF2a and tetranor 12-HETE, EPA-derived 9-HODE, DGLA-derived 13,14-dh-PGE1, and DHA-derived 10(11)-EpDPE species (Fig. 8D).

Interestingly, from the volcano plots, 9-HODE (LA-derived) was significantly higher in M ND SVF compared to M HFD and in F HFD in comparison to M HFD (main effect of sex, *P* < 0.05; supplemental Fig. S2J). 13(14)-EpDPE (DHA-derived) and 17(18)-EpETE (EPA-derived) were significantly lower in F HFD than F ND SVF and M HFD SVF (main effect of sex, *P* < 0.05 and *P* < 0.01; supplemental Fig. S2K, L), while 13(14)-EpDPE was higher in M HFD SVF than M ND and F HFD (main effect of sex and diet, *P* < 0.01 and *P* < 0.05; supplemental Fig. S2L). These results suggest that both sex and diet play a role in the generation of the many oxylipin derivatives.

Overall, the most significant changes were in total AA-derived metabolites and the number of upregulated pro-inflammatory AA derivatives in M HFD GWAT SVF (77, 78) when compared to M ND (Fig. 8A and supplemental Table S3B). F HFD GWAT SVF also had an increase in total AA-derived metabolites compared to F ND GWAT SVF (Fig. 8A) and several AA-derived metabolites although the type of oxylipin produced differed (supplemental Table S3A, B) from M HFD GWAT SVF. This suggests that a balance of both omega-3 and omega-6 metabolites may play a role in the sex differences in GWAT ATM lipid metabolism (13, 14, 15, 64).

### Sex differences in PUFA and oxylipin gene pathways in ATM correlate with oxylipin lipidomics

**Fig. 9:**
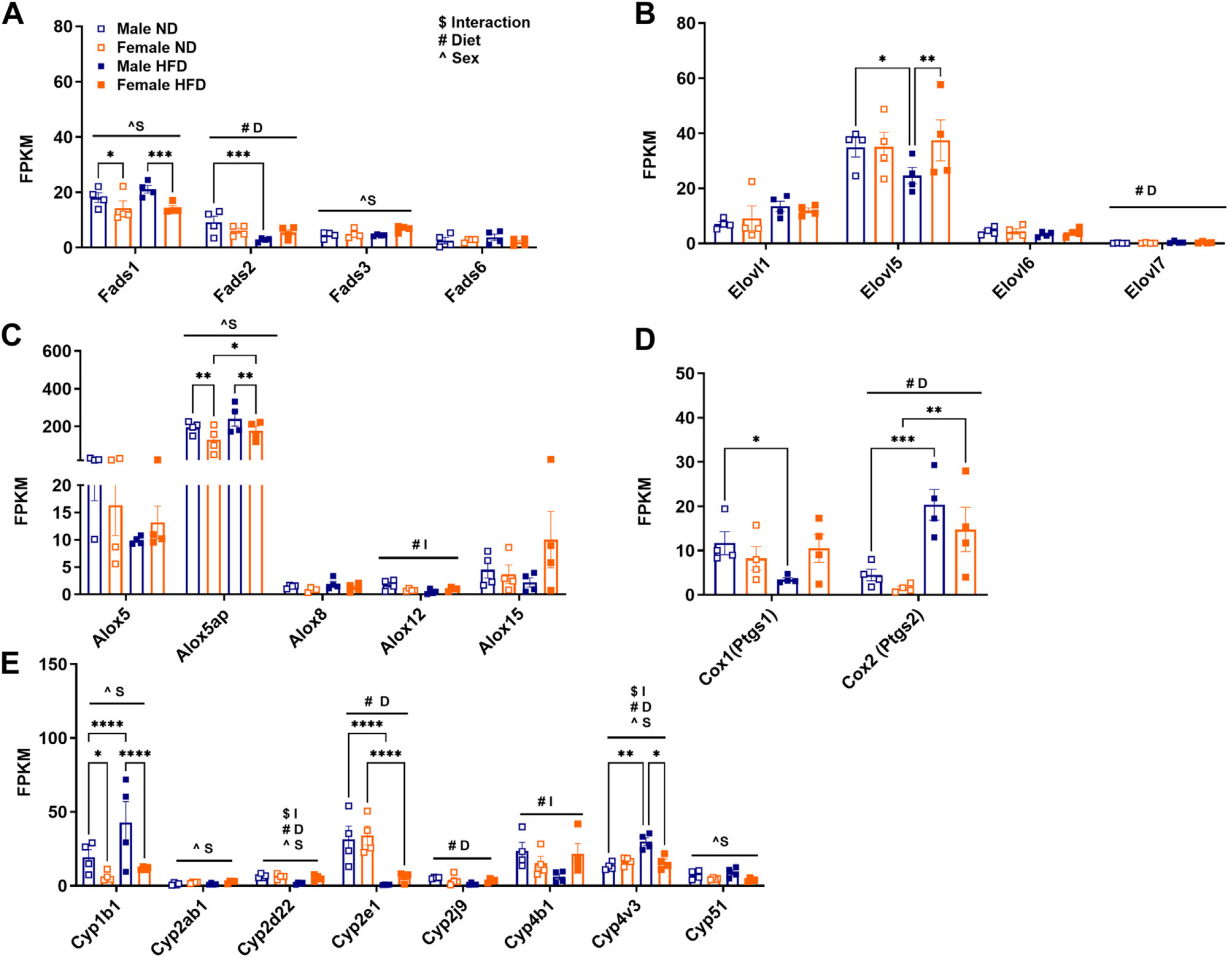
Analysis of PUFA and oxylipin pathway genes in GWAT ATMs with scRNA sequencing. FPKM counts from single-cell RNA-seq data showing gene expression of (A) *Fads*, (B) *Elovl*, (C) *Alox*, (D) *Cox (Ptgs)*, (E) *Cyp*. N = 4 per group. Data were analyzed by 2-way ANOVA followed by Fisher’s LSD multiple comparisons test. Data shown as average ± SEM. ∗*P* < 0.05, ∗∗*P* < 0.01, ∗∗∗*P* < 0.005, ∗∗∗∗*P* < 0.0001. Figures are marked for significance of main effects (ˆ Sex or # Diet) and $ Interaction.

PUFAs in ATMs can be synthesized from LA and alpha-LA by the action of fatty acid elongase (*Elovl*) and desaturase (*Fads*) enzymes to generate downstream pro-inflammatory mediators such as oxylipins through COX or prostaglandin synthase, lipoxygenase, and cytochrome P450 pathways (79, 80, 81, 82). To determine the origin of the sex differences in PUFA and oxylipins, we interrogated pathway genes from sorted GWAT ATMs (PI^-^, CD45^+^, CD64^+^) from male and female lean and obese mice for RNA-seq. Previously, we had reported several pathways altered between male and female ATMs leading to differences in metabolism, inflammatory polarization, and function (60). Sex differences were specifically observed in both ATM fat storage (regulation of lipolysis, carbohydrate digestion) and inflammation (hematopoietic cell lineage, chemokine receptors, cytokine–cytokine interactions) pathways (60). With the lipidomics findings, we focused on PUFA and oxylipin pathway genes (Fig. 9). Desaturase genes *Fads1* and *Fads3* were differential by main effect of sex (*P* < 0.05), while *Fads2* was different among the groups for main effect of diet (*P* < 0.05) (Fig. 9 and supplemental Table S4). Elongase gene, *Elovl7* was different for the main effect of diet (*P* < 0.05) (Fig. 9 and supplemental Table S4). Lipo-oxygenase gene, *Alox5ap* was differential by sex (*P* < 0.05), while prostaglandin synthesizing or cyclo-oxygenase gene, *Cox (Ptgs)* was differential by sex (*P* < 0.005) (Fig. 9 and supplemental Table S4). Among the *Cyp* genes, *Cyp1b1*, *Cyp2ab1*, and *Cyp51* were different among the groups for main effect of sex (*P* < 0.05), while *Cyp2e1* and *Cyp2j9* were significantly different among the groups for main effect of diet (*P* < 0.005 and *P* < 0.05). On the other hand, *Cyp2d22* and *Cyp4v3* were significantly different among the groups for main effects of both diet and sex (diet and sex interaction, *P* < 0.05) (Fig. 9 and supplemental Table S4). supplemental Table S4 shows the statistical *P* values for 2-way ANOVA for main effects of diet and sex and interaction for all RNA-seq genes listed in Figure 9. These results suggest that both diet and sex influence the generation of various oxylipin mediators. To confirm validity of data from RNAseq analysis of the major oxylipin generating enzyme genes, we performed gene expression of *Alox, Cox1 (Ptgs1) Cox2 (Ptgs2)*, and some *Cyp* genes (supplemental Fig. S3A–G) in HFD-fed male and female GWAT SVF.

## Discussion

Storage of excess lipids is a crucial homeostatic mechanism, but dysfunctional lipid metabolism drives cell-specific inflammation, which is a key component in the development of metabolic syndrome. While systemic lipid metabolism can influence bone marrow–derived inflammatory cells (33, 64), tissue-specific lipids can influence local immune cells driving insulin resistance. The relevance of this phenomenon is confirmed by several clinical reports demonstrating ATM infiltration and activation in the white adipose tissue of obese humans (16, 18, 20). However, sex hormones alone do not fully explain the sexual dimorphism on lipid storage and metabolism as these changes were only marginally normalized by gonadectomy, suggesting a major role for diet in dictating inflammatory responses. Our findings of sex differences in lipid species in male and female mice visceral AT depots with obesity suggests that both diet and hormonal fluctuations are equally responsible in driving changes in adipose lipid composition.

The untargeted lipidomic study in HFD-fed GWAT from M Sham, F Sham, M GX, and F GX demonstrates a sex regulation of many lipid classes such as DG, CE, FFA, FAHFA, and BMP with their levels being higher in obese males than females. Higher levels of these lipid classes have been correlated to metabolic defects (83, 84, 85) that male mice might be prone to in obesity, while only a few lipid species such as acylcarnitines, palmitic acid, and AA were different by GX. Higher acylcarnitine levels that activate proinflammatory pathways (66) and indicative of inefficient β-oxidation were significantly different by GX in ovariectomized female GWAT, suggesting a protective effect of female sex hormones to clear excess FAs as observed in other studies. When we investigated the effects of sex and GX on saturation and unsaturation of FAs, we observed that total MUFA and PUFA including oleic acid, linoleic acid, eicosenoic acid, eicosadienoic acid, and EPA were significantly different by sex (higher in males) while the SFA palmitic acid was significantly different by GX. These results suggest sex differences in MUFA and PUFA metabolism as examined in other studies (86). On the other hand, AA content was significantly different by both sex and GX. PUFAs such as AA are synthesized during normal cell homeostasis or are activated in conditions of stress, functioning as activators of counterregulatory, anti-inflammatory, and proresolution mechanisms (87). Sex differences observed in the lipid composition of many lipid classes are therefore consistent with the sex-specific differences in susceptibility to metabolic disorders (88) but here we identify some similarities in metabolism by sex.

Sex differences in the relative levels of PL classes such as BMP, PG, PEtOH, and PMeOH were also observed. In obesity as the AT is remodeled, these PL classes have been strongly associated with meta-inflammation and apoptosis (89), suggesting that alterations in PL species (15, 86, 90) might be significant for inflammation in obese males. In the GX groups, castration led to a decrease in PS and PEtOH levels with an increase in PA and PMeOH. On the other hand, ovariectomy led to an increase in PC and PMeOH levels and decrease in PE. These changes suggest a potential role for male sex hormones on PL remodeling in the GWAT. Among the species, PE (32:1), LNAPE (N-33:2), and LNAPE (N-38:2) were downregulated in M Sham HFD GWAT compared to female, while PI(36:4), PC(36:3), and PC(38:3) were upregulated compared to M GX HFD GWAT. F Sham GWAT when compared to F GX GWAT showed PE (35:0), PE (36:5), and LPC (14:0) to be upregulated. LNAPE species are Lyso-PE that have been associated with sex hormone alterations in other studies (91). Interestingly, total PC and PE were significant for interaction by sex and GX and the ratio of PC/PE was higher in male GWAT than female GWAT but decreased with castration while being increased with ovariectomy. Notably, PC/PE ratio determines the regulation of lipid droplet synthesis and hydrolysis within the adipocyte (92). In the AT, lower ratios suppressed de novo lipogenesis leading to healthy expansion of adipocytes (93, 94, 95), usually observed in females (1, 88). Overall, these findings are aligned with prior findings on adipocyte expansion by sex and GX (64, 88, 94).

The sex dimorphism of PL may be attributed to the differential expression and activity of PL metabolic enzymes between the sexes (96). One possible mechanism for the shift in PL species would be changes in SCD1 activity (97). Using the ratio of stearic acid/oleic acid (18:1/18:0) as a marker of SCD1, higher SCD1 activity was observed in male GWAT and with ovariectomy implying an effect of the male sex hormone on the SCD1 pathway. High SCD1 expression is correlated with metabolic diseases such as obesity and insulin resistance, whereas low levels are protective against metabolic disturbances (65). High SCD1 activity in males might significantly upregulate very long acyl chain containing PC, PE, PG, and PS lipid species that might generate greater proportions of toxic lipid intermediates such as SM and Cer (98). Consistent with this, sphingolipids such as SM and PE-Cer were also found to be sex-regulated with higher levels in male obese GWAT. Ceramides promote insulin resistance by lowering the prenol lipid CoQ in the mitochondria (99, 100, 101). CoQ content was altered by sex and GX with higher levels in F Sham and F GX, suggesting a role for sex hormones in CoQ homeostasis and inflammation. Although the relevance of these lipid species need to be validated with further experiments, this might explain higher thermogenic capacity in female AT and subsequent protection against obesity-related metabolic diseases in female obese mice (99, 102).

Another class of PL, the ether-linked PL or PL-O, the major end product of the ether lipid synthesis pathway (73) have been implicated in cardiovascular disease (103, 104) and associated with inflammation in obesity-related metabolic disease (105, 106). Previous studies have suggested regulatory roles of PL-Os in mitochondrial oxidative metabolism in AT (107). Many species of PL-O, PC-O, PE-O, LPC-O, and LPE-O were found to be altered especially in the male HFD GWAT compared to female. Several PE-O species also showed characteristic differences in F GX HFD GWAT, suggesting an influence of female sex hormones on their composition as observed in other studies (98, 106). In another lipidomic study, Yang *et al.* showed that lipectomy in mice increased inflammation in both the testis and ovary on the side of the lipectomy as well as altered the levels of free carnitine, PC, and PL, suggesting the role of visceral AT lipid metabolism on reproductive health (108). Overall, our untargeted lipidomic analysis showed that sex hormones can modulate lipid composition of AT, suggesting a need for sex-based strategies for clinical intervention studies in obesity. However, further studies through activation and inhibition of certain lipid target genes are required to validate the shifts in the many lipid species and the effect of these alterations in AT metabolism, which is a limitation of this study.

PUFAs are the precursors of lipid mediators associated with inflammatory responses as well as resolution of inflammation in ATMs (109). Therefore, in addition to direct adipocyte-related metabolic and adipogenesis effects of these lipid mediators, ATMs can participate in the physiological and pathological remodeling of AT (110, 111, 112) via modulating adipogenesis (113), regulating angiogenesis (114) and lipids themselves through lipid uptake, trafficking, and metabolism. Targeted lipidomics of the GWAT SVF FFA and PUFA metabolites showed a significantly higher ratio of omega 6: omega 3 PUFA substrates in the male HFD SVF fraction than females. Flow cytometry analysis further confirmed increased lipid uptake in male pro-inflammatory ATMs as well as a greater expression of *Mcp1*. Thus, adipocyte-macrophage crosstalk through pro-inflammatory cytokines and oxylipins such as PGs, TXs, and LTs in ATMs might impact its own phenotype and/or promote overall neighboring adipocyte metabolic health in a sexually dimorphic manner (115, 116).

PPARγ, a major regulator of adipogenesis, is known to be a direct target of PUFA metabolites (117). PPARγ when activated by ligands such as linoleic acid, linoleic acid derivatives such as 9- and 13-HODE, AA derivative such as 15-deoxy-12,14-prostaglandin J2 (PGJ2), as well as 8-S-HETE promotes adipocyte differentiation (118, 119, 120). 9-HODE increased FABP4 expression in THP-1 monocytes and macrophages through PPARγ activation (121). From the targeted lipidomics of SVF, Cyp2b-generated 9-HODE showed sex regulation with increased levels in the obese female GWAT SVF, suggesting possible regulated control over adipogenesis in obese females. In Cyp2b-null mice studies, serum lipids increased, especially in females, after 9-HODE treatment demonstrating sex differences (122). 17,18-EpETE that showed both effects of sex and diet was found in other studies to induce brown and beige adipocyte thermogenesis, with increased expression of thermogenic marker gene UCP1 in differentiated stromal vascular cells (123). COX products, such as PGE2, PGF2α, and leukotriene B4 inhibit adipocyte development (124, 125). Interestingly, PGE2 levels were observed to be significantly upregulated in obese male and female GWAT SVF compared to their lean counterparts (supplemental Table S3B, D. However, PGJ2 was only significantly higher in the obese female GWAT SVF (supplemental Table S3D). Hence, the balance between PGE2 and PGJ2 signaling might be central to protection from metabolic alterations in female obesity. Our study demonstrates that in addition to the number of AA-derived mediators being lower in proportion in the female obese GWAT SVF than males, both omega-3 (alpha-Linolenic) and omega-6 (LA)-derived metabolites were upregulated in F HFD GWAT SVF comparatively. Male obese GWAT SVF exhibited a preference for AA metabolism linked to inflammation while obese female GWAT preferred metabolism of DHA towards more of a resolution phenotype implying sexual dimorphism in the inflammatory response in obesity. The compelling differences in oxylipin mediators suggest that dietary PUFAs influence macrophage immune responses modulating their ability to polarize, interact with other immune cells and adipocytes.

While these studies highlight the role of diet and sex dimorphism in HFD-induced obesity and dependence on sex hormones, certain limitations highlight areas for future studies. One of the limitations of the study is the bulk nature of the GWAT studies and that the GWAT SVF fraction was not sorted into leukocytes for targeted lipidomics, which may further help us to better identify the role of AA-derived mediators in sex-dependent inflammatory response directly. Another limitation of the study is the lack of a targeted PUFA metabolome study in adipocytes compared to ATMs which would also help to better understand the significance of adipocyte–ATM interaction. While a localized focus on visceral adipose tissue is important because of the local inflammation driving insulin resistance, fat depots expand in different regions by sex, and there is heterogeneity in inflammatory responses in depots, inclusion of additional adipose depots in future studies may provide a more comprehensive view in the field. Future targeted lipidomics in depot and cell-specific evaluations would help to confirm lipid profiles from the untargeted analysis and further determine which lipid species specifically can be modified to create a more regulatory environment without impaired inflammation. To validate sex and diet effects of oxylipin mediators, future studies with targeted inhibition or activation of Cox, Lox, and Cyp genes are required in both male and females. Lastly, given findings that gonadectomy failed to completely normalize the lipid composition to control conditions, evaluations of cell-specific and developmental sex hormone receptor KO mice (androgen receptor knock out or estrogen receptor KO) or androgen treatment would be alternative methods to investigate sex hormone fluctuations on lipid metabolism and inflammatory metabolites.

Understanding the mechanisms that give rise to sex differences in AT function in obesity are critical for directing strategies for sex-specific management. Our findings of sex differences in lipid metabolism and impaired inflammation provide an insight into sex-specific control of metabolic pathways that may alter disease risk factors. These findings of sexual dimorphism in stored lipid species and PUFA-derived mediators both emphasize differences in individual dietary lipid metabolism and inflammatory responses that require future investigations.

## Data availability

The data that supports the findings of this study will be available upon request from the corresponding author (ksinger@umich.edu).

## Supplemental data

This article contains supplemental data.

## Conflict of interest

The authors declare that they have no conflicts of interest with the contents of this article.
