# Supplementary material for: Sex differences in the lipid profiles of visceral adipose tissue with obesity and gonadectomy
Source: J Lipid Res. 2025 Apr 15;66(5):100803. doi: 10.1016/j.jlr.2025.100803 (PMC12144442; doi:10.1016/j.jlr.2025.100803)
Supplement: Supplemental data [file mmc1.docx]

SUPPLEMENTAL INFORMATION

**Sex differences in lipid profiles of visceral adipose tissue with obesity and gonadectomy**

Mita Varghese^1^, Rajendiran Thekkelnaycke^2^, Tanu Soni^2^, Jiayu Zhang^2^,

Krishnarao Maddipati^3^ and Kanakadurga Singer ^1^

^1^Department of Pediatrics, Michigan Medicine, University of Michigan, Ann Arbor, MI, USA

^2^Michigan Regional Comprehensive Metabolomics Resource Core, University of Michigan,

Ann Arbor, MI, USA

^3^Department of Pathology, Wayne State University, Detroit, MI, USA.

**Supplementary Table S1. List of internal standards used in untargeted lipidomic analysis.**

| **Item #** | **Supplier** | **Product #** | **CAS-Number** | **Description** |
| --- | --- | --- | --- | --- |
| 1 | Avanti | 855676P | 50930-23-9 | 17:0 Lyso PC |
| 2 | Avanti | 850360P | 70897-27-7 | 17:0 PC |
| 3 | Avanti | 830756P | 140219-78-9 | 17:0 PE |
| 4 | Avanti | 830456P | 799268-52-3 | 17:0 PG |
| 5 | Avanti | 860517P | 67492-16-4 | 17:0 Ceramide |
| 6 | Avanti | 860585P | 121999-64-2 | 17:0 SM |
| 7 | Avanti | 840028P | 799268-51-2 | 17:0 PS |
| 8 | Avanti | 830856P | 154804-54-3 | 17:0 PA |
| 9 | Sigma-Aldrich | T2151 | 2438-40-6 | 17:0 TG |
| 10 | Sigma-Aldrich | SMB00506 | 5638-14-2 | 17:0 MG |
| 11 | Sigma-Aldrich | 68633 | 372490-74-9 | 19:0 DG |
| 12 | Avanti | 700186 | 24365-37-5 | 17:0 Cholesteryl ester |
| 13 | Sigma-Aldrich | 616966 | 241157-04-0 | Tri(palmitin-d31) |
| 14 | Avanti | LN1502 | 1246304-59-5 | 17:0-20:4 PI |

**Supplementary Table S2. Primer sequences for qRT-PCR.**

|  | **Forward Primer (5’ to 3’)** | **Reverse Primer (5’ to 3’)** |
| --- | --- | --- |
| *Arbp* | AGATTCGGGATATGCTGTTGGC | TCGGGTCCTAGACCAGTGTTC |
| *Mcp1* | TTAAAAACCTGGATCGGAACCAA | GCATTAGCTTCAGATTTACGGGT |
| *Fads1* | CCAGCTTTGAACCCACCAA | CATGAGGCCCATTCGCTCTA |
| *Fads2* | TCAAAACCAACCACCTGTTCTTC | ACACTCTATCACTGGCATCC |
| *Cox1* | TAGGGTGGCGTCCAGAACA | GGTCACTCCTCCCAGAATAACTG |
| *Cox2* | ACACTCTATCACTGGCATCC | GAAGGGACACCCTTTCACAT |
| *Alox12* | TCCCTCAACCTAGTGCGTTTG | GTTGCAGCTCCAGTTTCGC |
| *Alox15* | CTCTCAAGGCCTGTTCAGGA | GTCCATTGTCCCCAGAACCT |
| *Cyp2j* | TCTGGGAAGCACTCCATCTCA | CCCTGGTGGGTAGTTTTTGG |


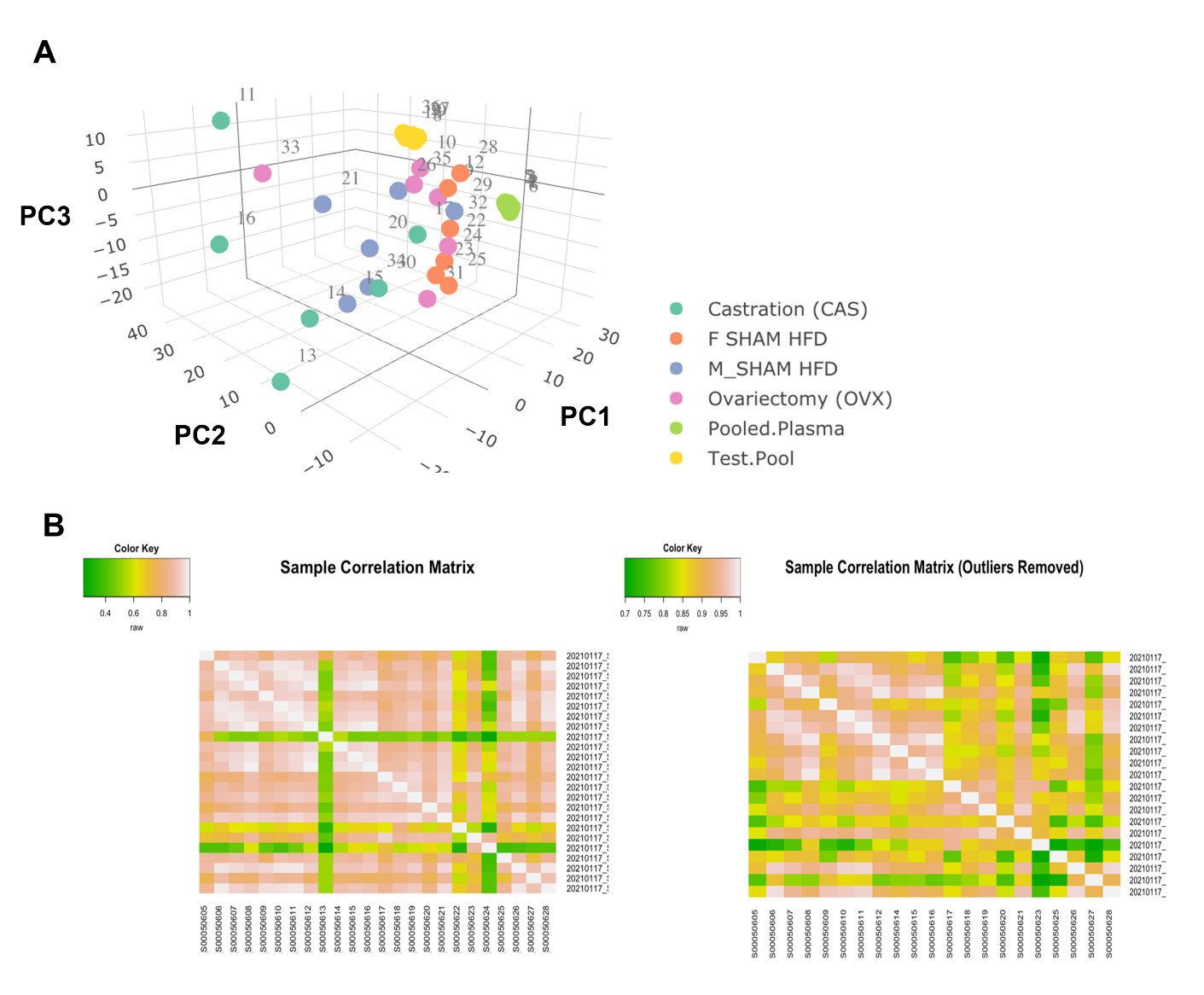


**Supplementary Figure S1**

**Supplementary Fig S1. Exploratory data analysis of untargeted lipidomics data. (**A) PCA plot of M Sham HFD, F Sham HFD, CAS (M GX HFD) and OVX (F GX HFD). N = M Sham HFD (6), F Sham HFD (6), M GX HFD (6) and F GX HFD (6). PCA analysis was performed by combining the positive and negative mode datasets. (B) Sample correlation matrix. This analysis was performed to detect and omit sample outliers resulting in inclusion of N = M Sham HFD (6), F Sham HFD (5), M GX HFD (5) and F GX HFD (5) for further analysis. HFD, high-fat diet; CAS, castration; OVX, ovariectomy; GX, gonadectomy.


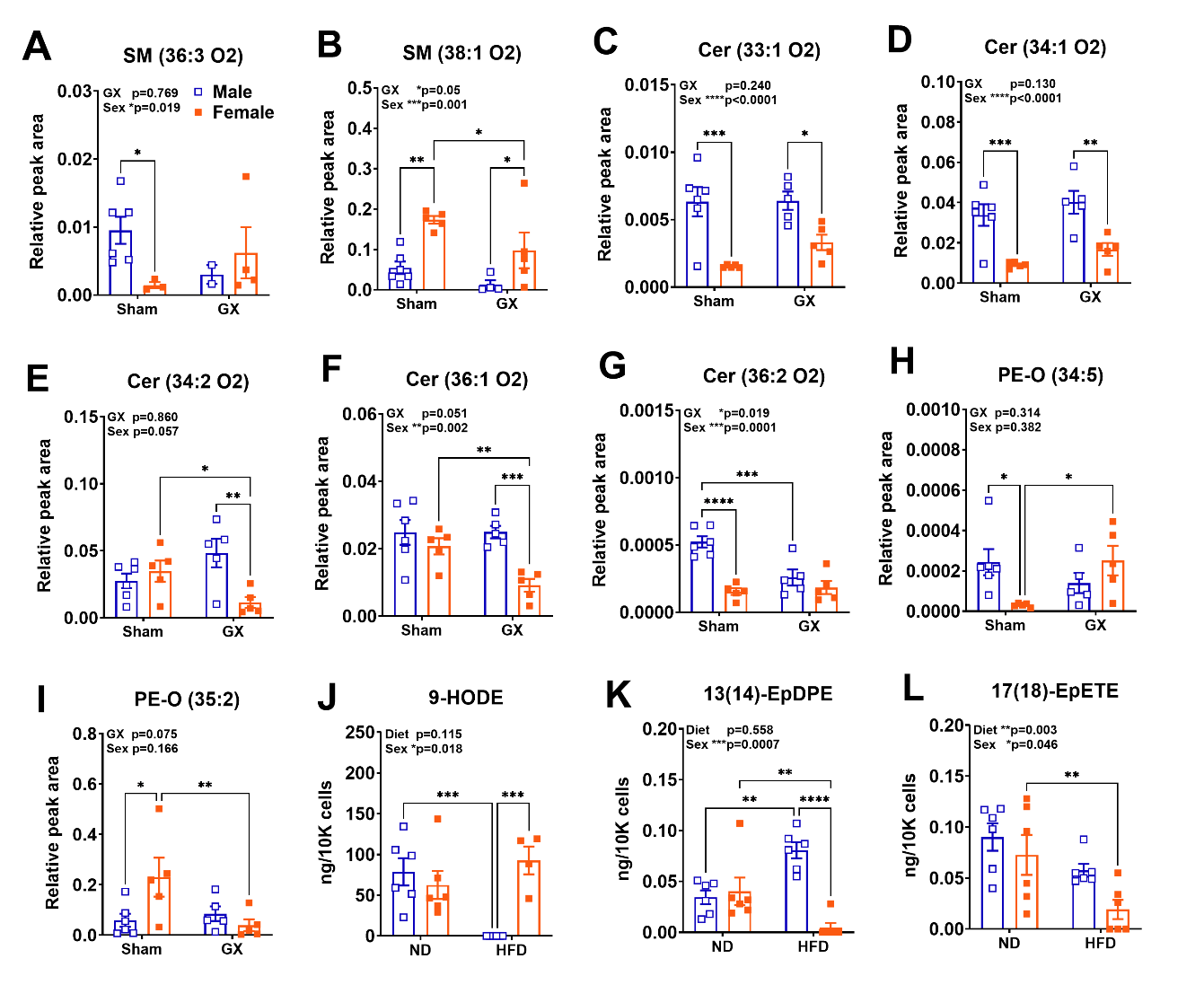
 **Supplementary Figure S2**

**Supplementary Fig. S2. Lipid species prominently altered in GWAT SVF volcano plots. Untargeted lipidomics (A-I), targeted lipidomics (J-L).** Plots depicting relative abundance as peak area of (A) SM(36:3; O2) (B) SM(38:1; O2) (C) Cer (33:1;O2) (D) Cer (34:1;O2) (E) Cer (34:2;O2) (F) Cer (36:1;O2) (G) Cer (36:2;O2) (H) PE-O (34:5) (I) PE-O (35:2) and oxylipins as ng/10k cells of (J) 9-HODE (K) 13(14)-EpDPE (L) 17(18)-EpETE Lipid species that were significantly different were chosen from corresponding SM, Cer, PL-O and GWAT SVF volcano plots. Data analysis was performed by 2-way ANOVA accounting for sex and GX (A-I) or sex and diet (J-L) followed by *post hoc* analysis for multiple comparisons with Fishers LSD test. Data shown as average ± SEM. ∗p < 0.05, ∗∗p < 0.01, ∗∗∗p < 0.001, and ∗∗∗∗p < 0.0001. A-I, N = M Sham HFD (6), F Sham HFD (5), M GX HFD (5) and F GX HFD (5). J-L, N= M ND (6), M HFD (6), F ND (6) and F HFD (6). GWAT, gonadal white adipose tissue; HFD, high-fat diet; GX, gonadectomy; SM, Sphingomyelin; Cer, Ceramide; PE-O, Ether-linked phosphatidylethanolamine; HODE, hydroxyoctadecadiecnoic acid; EpDPE, epoxy-docosapentaenoic acid; EpETE, epoxy-eicosatetraenoic acid.

**Supplementary Table S3. From Fig. 8 volcano plots.**

**S3A. Metabolites up in M ND GWAT SVF from volcano plot of M ND vs M HFD GWAT SVF (from Fig. 8B).** Arachidonic acid (AA), Docosahexaenoic acid (DHA), Eicosapentaenoic acid (EPA), Eicosadienoi acid (EDA), Dihomo-g-linolenic acid (DGLA), Docosapentaenoic acid (DPA), Linoleic acid (LA), alpha-linolenic acid (a-LA). M, male; F, female; ND, normal diet; HFD, high-fat diet; GWAT, gonadal white adipose tissue; GX, gonadectomy; SVF, stromal vascular fraction.

| **Metabolites up in M ND (M ND vs M HFD)** | |
| --- | --- |
| **Metabolite** | **Derived from** |
| 9-HODE | LA |

**S3B. Metabolites up in M HFD GWAT SVF from volcano plot of M ND vs M HFD GWAT SVF (from Fig.8B)**

| **Metabolites up in M HFD (M ND vs M HFD)** | |
| --- | --- |
| **Metabolite** | **Derived from** |
| 6-keto PGF1a | AA |
| 15-keto PGF2a | AA |
| 12-HHTrE | AA |
| PGF2a | AA |
| LXA4 | AA |
| 11-HETE | AA |
| iPF-VI | AA |
| D12-PGJ2 | AA |
| 15-HETE | AA |
| 8-HETE | AA |
| 5-HETE | AA |
| PGE2 | AA |
| 12-OxoETE | AA |
| TXB2 | AA |
| 8(S),15(S)-DiHETE | AA |
| 11(12)-EpETrE | AA |
| 5(S),15(S)-DiHETE | AA |
| 14(15)-EpETE | AA |
| 13,14-dh-15k-PGE2 | AA |
| 5-HETrE | AA |
| 9-HETE | AA |
| 8-isoPGF2a & 11bPGF2a | AA |
| 12-HETE | AA |
| 8-HETrE | AA |
| 5-oxoETE | AA |
| 14(15)-EpETrE | AA |
| 11,12-DiHETrE | AA |
| PGA2 | AA |
| 14,15-DiHETrE | AA |
| 8,9-DiHETrE | AA |
| 12-OxoLTB4 | AA |
| 5,6-DiHETrE | AA |
| 15-keto PGE2 | AA |
| PGD2 | AA |
| 5(S),12(S)-DiHETE | AA |
| 11-HDoHE | DHA |
| 17-HdoHE | DHA |
| 16(17)-EpDPE | DHA |
| PDx(10S,17S-DiHDoHE) | DHA |
| 14-HdoHE | DHA |
| 10-HdoHE | DHA |
| 13-HdoHE | DHA |
| 7-HdoHE | DHA |
| 16-HdoHE | DHA |
| 8-HdoHE | DHA |
| 20-HdoHE | DHA |
| 13(14)-EpDPE | DHA |
| 12(13)-EpOME | DHA |
| 11-HEDE | Eicosadienoic acid |
| 15-HEDE | Eicosadienoic acid |
| 15-OxoEDE | Eicosadienoic acid |
| Bicyclo PGE2 | DGLA |
| D17-PGE1 | DGLA |
| 15-PGE1 | DGLA |
| 15-keto PGE1 | DGLA |
| PGD3 | EPA |
| 5,6-DiHETE(EPA) | EPA |
| 15d-D12,14-PGJ3 | EPA |
| 11-dh TXB3 | EPA |
| RvE2 | EPA |
| 15-HEPE | EPA |
| 13-HODE | LA |
| PD1 | DPA |

**S3C. Metabolites up in F ND GWAT SVF from volcano plot of F ND vs F HFD GWAT SVF (from Fig.8C)**

| **Metabolites up in F ND (F ND vs F HFD)** | |
| --- | --- |
| **Metabolite** | **Derived from** |
| 7(8)-EpDPE | DHA |
| 13(14)-EpDPE | DHA |
| 17(18)-EpETE | EPA |

**S3D. Metabolites up in F HFD GWAT SVF from volcano plot of F ND vs F HFD GWAT SVF (from Fig.8C)**

| **Metabolites up in F HFD (F ND vs F HFD)** | |
| --- | --- |
| **Metabolite** | **Derived from** |
| 6-keto PGF1a | AA |
| PGF2a | AA |
| 9-HETE | AA |
| 12-HHTrE | AA |
| 13,14-dh-15k-PGF2a | AA |
| 15-HETE | AA |
| 5(S),12(S)-DiHETE | AA |
| 11-HETE | AA |
| 8-HETE | AA |
| PGJ2 | AA |
| 5(S),15(S)-DiHETE | AA |
| 8,9-DiHETrE | AA |
| 5,6-DiHETrE | AA |
| 5-HETE | AA |
| 5-oxoETE | AA |
| 11(12)-EpETrE | AA |
| 12-OxoLTB4 | AA |
| 14,15-DiHETrE | AA |
| PGE2 | AA |
| 11,12-DiHETrE | AA |
| D12-PGJ2 | AA |
| 17-HdoHE | DHA |
| Maresin2 | DHA |
| 14-HdoHE | DHA |
| 16(17)-EpDPE | DHA |
| 10(11)-EpDPE | DHA |
| 10-HdoHE | DHA |
| 15-HEDE | Eicosadienoic acid |
| 11-HEDE | Eicosadienoic acid |
| 15®-PGE1 | DGLA |
| 8-HETrE | DGLA |
| RvE2 | EPA |
| 15d-D12,14-PGJ3 | EPA |
| 14(15)-EpETE | EPA |
| 9-OxoOTrE | a-Linolenic |
| 13-HOTrE | a-Linolenic |
| 9-HOTrE | a-Linolenic |
| 12,13-DiHOME | LA |
| 13-HODE | LA |
| PDx(10S,17S-DiHDoHE) | DPA |

**Supplementary Table S4. Figure 9 statistical analysis**

| **Gene** | **Diet** | **Sex** | **Interaction** |
| --- | --- | --- | --- |
|  |  |  |  |
| *Fads1* | 0.4195 | **0.0107** | 0.4199 |
| *Fads2* | **0.0209** | 0.895 | 0.0526 |
| *Fads3* | 0.1484 | **0.0279** | 0.1263 |
| *Fads6* | 0.7829 | 0.3679 | 0.3643 |
|  |  |  |  |
| *Elovl1* | 0.0906 | 0.9147 | 0.4893 |
| *Elovl5* | 0.4567 | 0.2261 | 0.2414 |
| *Elovl6* | 0.3133 | 0.6975 | 0.6589 |
| *Elovl7* | **0.0403** | 0.6915 | 0.5049 |
|  |  |  |  |
| *Alox5* | 0.0791 | 0.8058 | 0.2896 |
| *Alox5ap* | 0.1462 | **0.0498** | 0.9814 |
| *Alox8* | 0.2035 | 0.0758 | 0.9282 |
| *Alox12* | 0.0627 | 0.4058 | **0.0208** |
| *Alox15* | 0.4951 | 0.2407 | 0.1535 |
|  |  |  |  |
| *Cox1* | 0.2496 | 0.4800 | 0.0540 |
| *Cox2* | **0.0006** | 0.1961 | 0.6890 |
|  |  |  |  |
| *Cyp1b1* | 0.0838 | **0.0136** | 0.2623 |
| *Cyp2ab1* | 0.5270 | **0.0044** | 0.4799 |
| *Cyp2d22* | **0.0244** | **0.0021** | **0.0440** |
| *Cyp2e1* | **0.0002** | 0.5059 | 0.1193 |
| *Cyp2j9* | **0.0353** | 0.7346 | 0.0845 |
| *Cyp4b1* | 0.4895 | 0.3046 | **0.0400** |
| *Cyp4v3* | **0.0008** | **0.0019** | **0.0197** |
| *Cyp51* | 0.6984 | **0.0056** | 0.3206 |


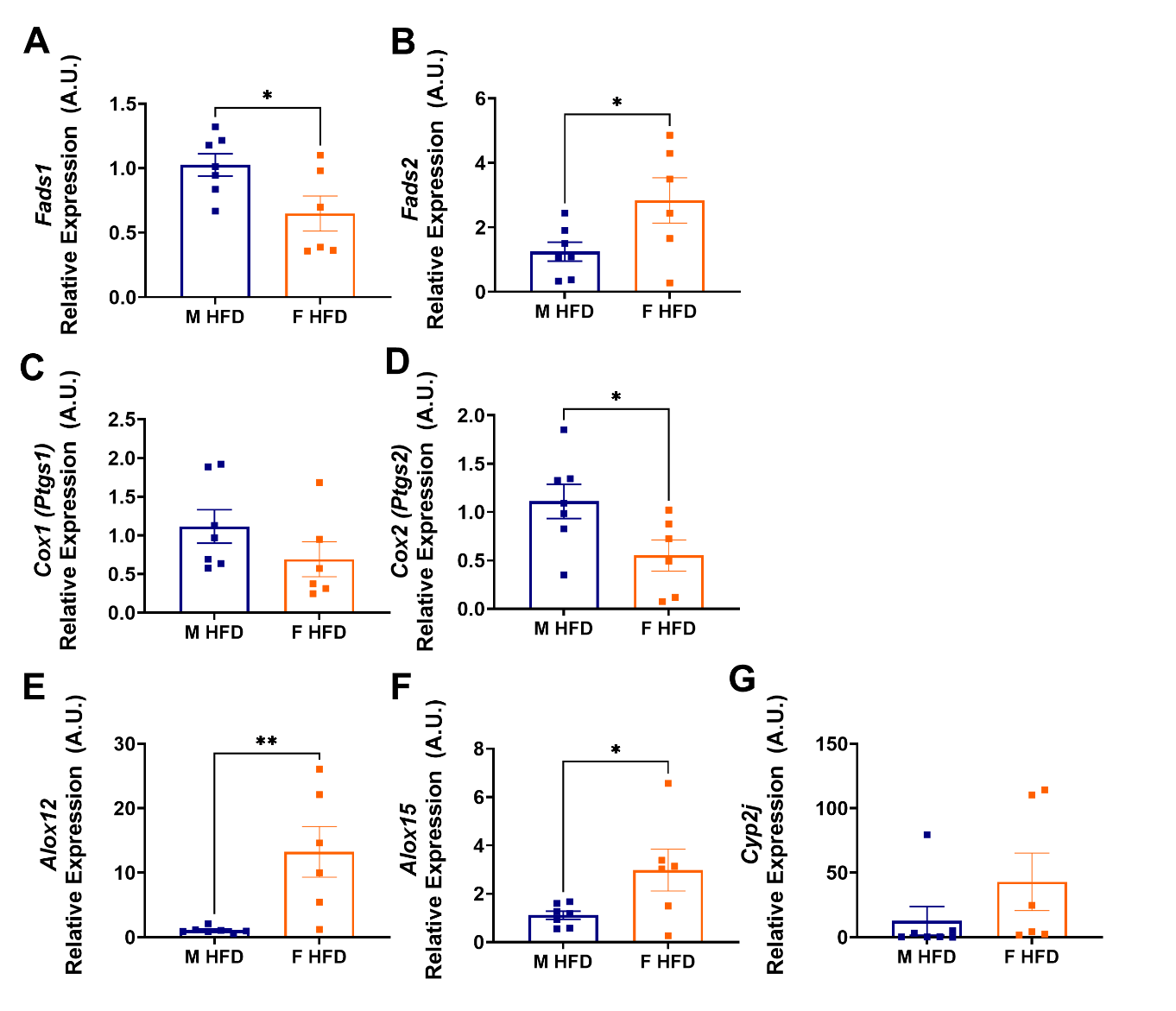


**Supplementary Fig. S3**

**Supplementary Fig. S3. qRT-PCR for validity of oxylipin genes from RNA sequencing of sorted ATMs.** Gene expression in male (M) and female (F) GWAT SVF of (A) *Fads1* (B) *Fads2* (C) *Cox1(Ptgs1)* (D) *Cox2 (Ptgs2)* (E) *Alox12* (F) *Alox15* (G) *Cyp2j.* Data analysis was performed by two-sample T-test. Data shown as average ± SEM. ∗p < 0.5, ∗∗p < 0.05, ∗∗∗p < 0.005, and ∗∗∗∗p < 0.0001. N= M HFD (7) and F HFD (6). GWAT, gonadal white adipose; SVF, Stromal vascular fraction.
